# Application of the Andersen-Newman model of health care utilization to understand antenatal care use in Kersa District, Eastern Ethiopia

**DOI:** 10.1371/journal.pone.0208729

**Published:** 2018-12-06

**Authors:** Gezahegn Tesfaye, Catherine Chojenta, Roger Smith, Deborah Loxton

**Affiliations:** 1 School of Public Health, College of Health and Medical Sciences, Haramaya University, Harar, Ethiopia; 2 Research Centre for Generational Health and Ageing, Faculty of Health and Medicine, University of Newcastle, Newcastle, NSW, Australia; 3 Mothers and Babies Research Centre, Faculty of Health and Medicine, University of Newcastle, Newcastle, NSW, Australia; Monash University, AUSTRALIA

## Abstract

**Background:**

In Ethiopia, the uptake of antenatal care services has been low. Moreover, there is less frequent and late attendance of antenatal care among women who attend. Using the Anderson-Newman model of health care utilization, this study identified factors that either facilitate or impede antenatal care utilization in Kersa district, Eastern Ethiopia.

**Method:**

A community-based cross-sectional study was conducted. A total of 1294 eligible women participated in the study. Data were collected using face to face interviews with a pre-tested structured questionnaire administered with a digital survey tool. Data were collected in a house to house survey of eligible women in the community. Bivariate and multivariate logistic regression analyses were used to examine the predisposing, enabling and need factors associated with antenatal care utilization.

**Result:**

Out of the 1294 respondents, 53.6% (95% CI: 50.8%, 56.3%) attended antenatal care at least once during their last pregnancy. Only 15.3% attended four or more antenatal care visits and just 32.6% attended prior to the 12^th^ week of gestation. Educational status, previous use of antenatal care and best friend’s use of maternal care were significant predisposing factors associated with at least one antenatal care visit. Type of *kebele*, wealth index and husband’s attitude towards antenatal care were significant enabling factors associated with at least one antenatal care consultation. Health Extension Workers providing home visits, perceived importance of ANC and awareness of pregnancy complications were significant need factor associated with at least one antenatal care consultation. Husband’s attitude towards ANC, head of the household, awareness of pregnancy complications, and history of abortion were predictors of attending four or more antenatal care visits.

**Conclusion:**

More than half of the women attended at least one antenatal care visit. A sizable proportion of women had infrequent and delayed antenatal care. Intervention efforts to improve antenatal care utilization should involve the following: improving women’s educational achievement, peer education programs to mobilize and support women, programs to change husbands’ attitudes, ameliorate the quality of antenatal care, increasing the Health Extension Worker’s home visits program, and increasing the awareness of pregnancy complications.

## Introduction

Maternal mortality remains a global public health priority, even in the era of Sustainable Development Goals (SDG) [1]. In many developing countries, including Ethiopia, obstetric complications that occur during pregnancy and parturition are the leading causes of maternal mortality among reproductive-aged women [2, 3]. Inadequate maternal care in resource-poor settings and insufficient utilization of available services, such as antenatal care (ANC), further aggravate the burden of maternal mortality [4]. Evidence has shown that providing timely and appropriate ANC leads to a reduction in maternal deaths [1, 5]. Antenatal care contributes to a reduction in maternal mortality, not only through facilitating prompt diagnosis and management of life-threatening obstetric complications but also through screening mothers who could potentially develop complications during childbirth [1, 6]. The ANC interventions offered during pregnancy include early detection and treatment of obstetric complications such as pre-eclampsia, antepartum haemorrhage, anaemia and nutritional problems. In developing countries, ANC also includes the provision of Tetanus Toxoid vaccination, early identification and treatment of sexually transmitted infections, provision of insecticide-treated bed nets, and prophylaxis medication for malaria [7].

The World Health Organization (WHO) recommends that all pregnant women access a minimum of four focused ANC check-ups with a skilled health worker [8, 9]. It was recommended that pregnant women should initiate the first ANC attendance during the first trimester [1, 9]. There has been good progress in ANC utilization in developing countries where the majority (80%) of women receive at least one ANC check-up [10]. However, only a small proportion (40%) of pregnant women in these countries have attended the minimum four ANC visits [11]. Furthermore, in sub-Saharan Africa, nearly three quarters (72%) of women initiate their first ANC check-up after the first trimester of pregnancy [10]. At the time this study was conducted, the WHO focused ANC model with a minimum of four visits and initiation of the first visit within the first trimester [9] was in place in Ethiopia and this research looked at the ANC practices of these recommendations.

In previous small-scale studies in Ethiopia [12–18], researchers demonstrated a fairly good level (70–88%) of at least one ANC consultation. Additionally, in the 2016 national Demographic and Health Survey report [19], almost two thirds (62%) of women received at least one ANC check-up for their most recent pregnancy. However, in many of these studies [13, 14, 16, 18] it was reported that the proportion of women who attended four or more ANC visits was below 40%. Furthermore, a similar finding was reported in the national survey [19] where only 32% received four or more ANC check-ups. In rural areas, the proportion of pregnant women who attended four or more ANC check-ups was only 27% compared to 63% in urban areas. Moreover, although evidence has shown that early initiation is associated with better maternal health outcomes [20, 21], nearly two thirds (64%) of women in Ethiopia delay their first ANC enrolment to the second or third trimester of pregnancy [22].

There is empirical evidence [12, 23–26] that socio-demographic, reproductive and obstetric factors as well as previous experience of service use were associated with poor utilization of ANC. Yet there is a need to examine these factors in a systematic way within one study, to permit identification of the most important barriers and facilitators to ANC uptake. The Andersen and Newman Behavioural Model (ANBM) for health service utilization provides a framework that permits systematic identification of factors that influence individual decisions to use (or not use) available health care services [27, 28]. According to the ANBM, *predisposing factors* are those socio-cultural characteristics of the individual that exist prior to their health condition, *enabling factors* reflect the means or logistics required to obtain the services, and *need factors* are the most immediate cause of health service use and reflect the perceived health status of the individual.

In order to uncover the contextual factors that affect the uptake of ANC, it is crucial to carry out studies based on a health model which addresses most aspects of personal decision making regarding health care utilization. Therefore, using the ANBM for health service utilization, the present study aims to identify the key factors that affect ANC utilization among reproductive-aged women in Kersa district, Eastern Ethiopia.

## Materials and method

### Study setting

A community-based cross-sectional study was conducted in Kersa district, Eastern Hararghe zone of Oromia regional state, in Eastern Ethiopia from June to August 2017. The district capital is Kersa town, which is located 486 kilometres from the capital city, Addis Ababa. According to the population projection for Ethiopia published by the Central Statistical Agency in 2014, the district has an estimated total population of 205,628. The population is predominantly rural (92%). The district has 38 *kebeles* of which three are urban and 35 are rural [29, 30]. The *kebele* is the lowest administrative unit in Ethiopia consisting of around 1000 households, or an approximate population of 3000 to 5000 [31]. The district has 24 Health and Demographic Surveillance System (HDSS) *kebeles* and 14 non-HDSS *kebeles*. HDSS is a platform to regularly follow the health and demographic characteristics of a community residing in a distinct geographic area. It monitors new health threats, tracks the change in population number through fertility and migration rates, and measures the effect of interventions on communities [32, 33]. All *kebeles* have access to non-asphalt roads, though the terrain is mountainous in the majority of the rural *kebeles*. The district has seven health centres, thirty four health posts and eight private pharmacies at different locations within the district. In each *kebele*, there are two Health Extension Workers (HEWs) providing health promotion activities. According to the information from the district health office, the health coverage (physical accessibility of health facilities) of the district is more than 80% [34].

### Population

The study population was all reproductive-aged women in the Kersa district who gave birth in the three years prior to the survey, regardless of the birth outcome. Women who had lived in the district for more than six months, and delivered their most recent baby after 28 weeks of gestation were included. Women who did not volunteer to participate in the study, were critically ill and physically or mentally disabled during the data collection period, were excluded from the study.

### Sample size and sampling procedure

The sample size (n = 1320) was primarily determined for a broad study on the level of maternal health service uptake and associated factors in Kersa district, Eastern Ethiopia. A total of ten *kebeles* (seven from areas where HDSS is conducted and three from non-HDSS areas) were included in the survey. Households with eligible women were identified using the HEW health management information system registration log sheet and the number of eligible women in each included *kebele* was determined. The total sample size of the study was proportionally allocated to each *kebele*. Respondents were selected through systematic random sampling techniques and invited to take part in the survey. When two or more eligible women were found within the selected household, one was selected by the lottery method and invited to do the interview.

### Measurement variables

#### Outcome variables

***At least one ANC attendance*:** Women who have attended at least one ANC check-up during their most recent pregnancy as reported by the participant.

***Four or more ANC attendance*:** Women who attended four or more ANC visits during their last pregnancy as reported by the participant.

#### Predictor variables

The predictor variables were conceptualized based on the ANBM of health care utilization and grouped into three set of factors: predisposing, enabling and need factors as shown in Table 1.

**Table 1:** Predictor variables for PNC utilization and their operational definitions.

Variable category	Operational definition
***Predisposing factors***	
Maternal education	Formal schooling status starting from attending elementary school
Mass media availability	Presence of communication tools such as TV, radio or internet in the household
Telephone ownership	Ownership of mobile telephone at the household level
Education on maternal health	Receiving education on maternal health (such as the use of maternity service, and nutrition) from any source
Age at marriage	Completed in years at the time when the respondent first married
Age at first pregnancy	Completed in years at the time when the respondent first got pregnant
Birth order	Birthing order of the index child in the woman’s life
Previous use of ANC	Practice of ANC for any of the previous pregnancies in the woman’s life
Living in a model family	A woman who lives in a family which adopted and implemented the full health extension service packages in Ethiopia
Best friend’s use of maternal care	A friend who regularly shared the woman’s feelings, emotions, and opinions and various behavioural practices important in the woman’s life and uses services such as ANC, delivery care and PNC
***Enabling factors***	
Place of residence	*Urban* versus *rural* based on the *kebele* where the woman lives
Type of *kebele*	Based on the living site of the woman (*kebeles* under surveillance versus *kebeles* not under surveillance)
Wealth index	Produced from the existing variables (household assets ownership, household characteristics and access to utilities) from the data set through factor analysis using Principal Component Analysis (PCA)
Head of the household	A person who is responsible for heading the household
Decision making on household expenses	A person who decide on household matters including whether to visit health facilities during illness or emergency conditions
Husband’s attitude	Respondent’s judgement about the feeling of her husband towards ANC while she was pregnant
Social support	Type of help the woman received from her best friends during pregnancy, labour and post-delivery (prompted question)
***Need factors***	
Awareness of pregnancy complication	Respondents were asked whether they had knowledge of any dangerous pregnancy-related symptoms (such as bleeding)
Perceived importance of ANC	Women’s perception of how useful ANC attendance is for a healthy pregnant woman
Pregnancy intention	Women’s thinking on whether their last pregnancy was planned or not
HEW home visit	Receiving visit and health education by a HEW at woman’s home
History of abortion	Lifetime number of abortion coded as Yes or No

### Source of data and data collection methods

To obtain the data, we used house-to-house interviews with eligible reproductive-aged women using a structured questionnaire. The study tool for the survey was adapted from pertinent literature. The study tool was first prepared in English and subsequently translated into the local language (Oromiffa) to collect the data. The tool was re-translated back to English to check for consistency. A template of the study tool was prepared using an online survey tool (*Survey Gizmo*) and downloaded onto iPads for offline data collection. The data was collected by resident HDSS data collectors who have extensive experience in conducting interviews in both urban and rural *kebeles* using the iPads. The principal author (GT) and a supervisor closely monitored the overall data collection process.

#### Data quality control

The study tool was pre-tested on 65 women living in a neighbouring district. All required revisions were made to the study tool based on the pre-test. Experienced HDSS data collectors and a supervisor were recruited and deployed for the data collection. A two-day intensive training course was provided to the data collectors and the supervisor about the aim of the study and sampling procedures; data collectors also performed simulated exercises on how to interview respondents. To ensure correct inclusion of the participants, the student researcher made the random selection of the *kebeles*, was responsible for the proportional allocation of the samples, and carried out the random selection of the interviewees. We used iPads for data collection to avoid missing or incomplete responses. The supervisor cross-checked the completed responses on the iPads by repeating the interviews with 10% of the respondents to check for correct completion of valid responses. The responses were uploaded into the online survey tool on a daily basis and the lead author double-checked for any inconsistencies and gave feedback to the interviewers on daily basis.

### Data management and analysis

The data were directly exported from the digital survey tool into SPSS software version 22 for analysis. Before commencing data analysis, appropriate transformations were made on the variables and missing values were also managed as necessary. Descriptive statistics and appropriate measure of central tendencies were used to summarize the key variables. Variables with missing data due to the skipping nature of the question (*husband education* and *living in a model family*) were managed by re-coding the system missing value into an existing relevant category of the variable. Categories of some variables with negligible frequency, such as, “*don’t know*” or categories not relevant for a particular inferential test due to small cell value were recoded into another related category. Before fitting the full multivariate model, all of the variables were considered for the multicollinearity diagnostics and all showed no multi-collinearity with a variance inflation factor of less than five. Bivariate logistic regression analysis was conducted to examine the association between the predictor and outcome variables using the Crude Odds Ratio (COR) at a 95% confidence interval (CI). Factors that were significant with a p-value of less than 0.05 were retained for further consideration in three blocks of the multivariate logistic regression model. The three block models were built in such a way that, *model 1* contained only the *predisposing factors*, *model 2* included *predisposing* and *enabling factor*s, and *model 3* (the final full model) considered all three factors simultaneously (*S1 and S2 Tables*). In the final multivariate model, factors with a p-value of less than 0.05 were declared statistically significant [35]. Two separate multivariate models were fitted for primiparous and multiparous women for the “*at least one ANC*” outcome variable. The factors that showed statistically significant association in the multivariate logistic regression analysis were mapped into the three domains: predisposing, enabling and need factors to streamline the analysis using the ANBM.

### Ethics approval

The study was conducted after securing ethical approval from the Institutional Health Research Ethics Committee (IHREC) of the College of Health and Medical Sciences, Haramaya University, Ethiopia with approval number (IHRERC/129/2017) and the Human Research Ethics Committee (HREC) of the University of Newcastle, Australia with approval number (H-2016-0403). Informed verbal consent was obtained from each respondent before commencing interviews. The informed verbal consent procedure had been approved by both Ethics Committees. Participants read or listened to the Information Statement provided by the interviewer and it was expected that they understood its contents. Then, if they decided to participate, they informed the interviewer that they were happy to take part in the research, and this was taken as participant’s informed verbal consent. For respondents aged 15–18 years, we obtained informed consent from themselves as they were married, had had at least one birth, assumed social responsibility and are considered to be mature minors. The Ethics Committees had also approved the minor consent procedure for women between 15 and 18 years. The confidentiality of the respondents was ensured by avoiding personal identification details in the study tool.

## Results

### Socio-demographic characteristics

A total of 1294 women participated in the study. More than half (652, 50.4%) of the respondents fell within the age range of 25–34 years with a mean age of 27.4(±6) years. The majority of women were married (1277, 98.7%) with a large majority (1205, 94.2%) being in a monogamous marital union and the highest proportion belonging to the Oromo ethnic group (1274, 98.5%). The majority of respondents were Muslim (1253, 96.8%), housewives (1240, 95.8%), had never attended formal education (941, 72.7%) and had a husband who had never attended formal education (645, 50.4%) and who was engaged in farming (1156, 90.4%) (Table 2).

**Table 2:** Basic socio-demographic characteristics of the respondents, Kersa district, Eastern Ethiopia.

Variables	Frequency	Percentage
**Maternal age** (n = 1294)		
15–24	411	31.8
25–34	652	50.4
35–49	231	17.9
**Marital status** (n = 1294)		
Married	1277	98.7
Others *(Single*, *divorced*, *widowed and separated)*	17	1.3
**Type of marital union (**n = 1279**)**		
Monogamous	1205	94.2
Polygamous	74	5.8
**Ethnicity** (n = 1294)		
Oromo	1274	98.5
Others (*Amhara*, *Arab*)	20	1.5
**Religion** (n = 1294)		
Muslim	1253	96.8
Others *(Orthodox Christian and protestant)*	41	3.2
**Occupational status** (n = 1294)		
Housewife	1240	95.8
Government employee	24	1.9
Merchant	19	1.5
Farmer	11	0.9
**Educational status** (n = 1294)		
Never attended	941	72.7
Elementary (1–8)	267	20.6
Secondary (9–12)	61	4.7
Tertiary (12+)	25	1.9
**Wealth index** (n = 1294)		
Highest	258	19.9
Fourth	261	20.2
Middle	260	20.1
Second	258	19.9
Lowest	257	19.9
**Husband’s education (**n = 1279**)**		
Never attended	645	50.4
Elementary	438	34.2
Secondary	133	10.4
Tertiary	63	4.9
**Husband’s occupation (n = 1279)**		
Farmer	1156	90.4
Daily labourer	29	2.3
Government employee	65	5.1
Merchant	29	2.3
**Educated family member (n = 1294)**		
Yes	574	44.4
No	720	55.6

### Reproductive characteristics of respondents and knowledge about ANC

The majority of respondents (1114, 86.1%) were first married when they were under 18 years of age and 1155 (89.3%) became pregnant for the first time at or before 20 years of age. More than half (51.9%) of the women’s most recent births were a third child or less in birth order. Nearly two thirds (63.8%) of the women had been pregnant three or more times in their lifetime. Moreover, 820 (63.4%) of the women had experienced three or more deliveries in their lifetime. A total of 94 (7.3%), 95 (7.4%) and 264 (20.4%) had a history of abortion, still-birth and infant death respectively. Additionally, 917 (70.9%) women reported that their last pregnancy was intended, while for 377 (29.1%) of the women it was unintended.

**Fig 1:**
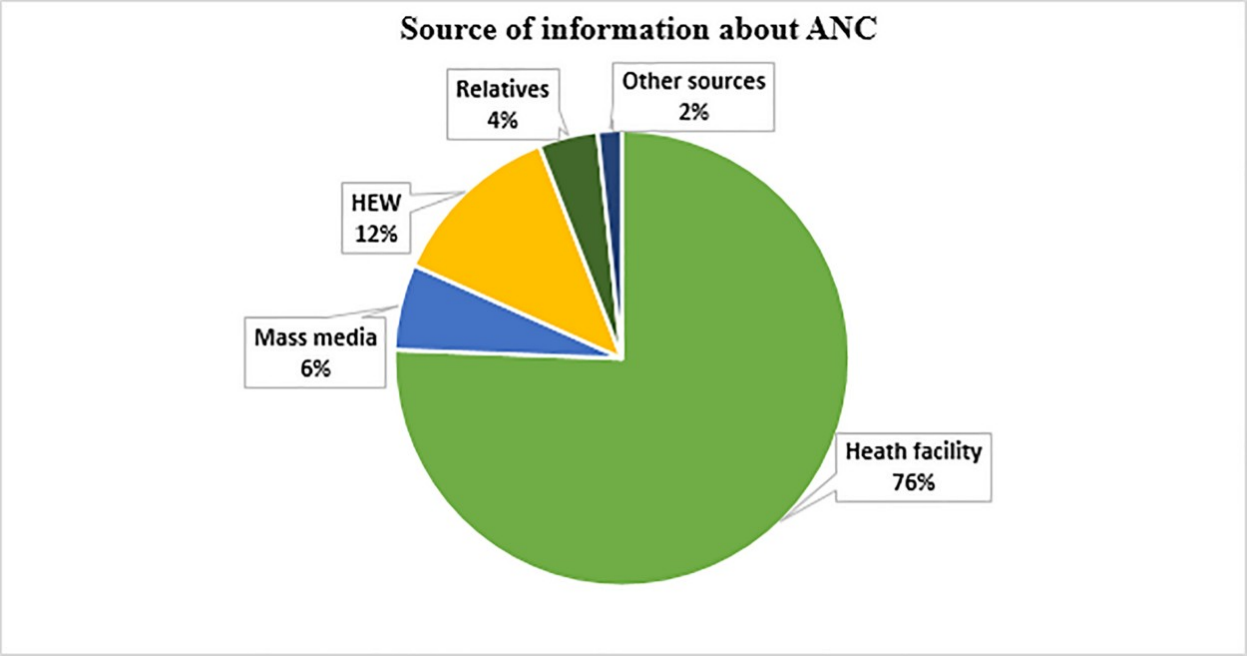
Sources of information about ANC among reproductive women in Kersa district, Eastern Ethiopia, 2017.

With regards to women’s knowledge of ANC, more than three-quarters (1000, 77.3%) had heard about ANC and 644 (49.8%) believed that ANC is beneficial for both the mother and the child. More than three quarters (76%) of the women had heard about ANC from a health facility, followed by a HEW (12%) (Fig 1). A total of 1242 (96%) respondents felt that pregnant women should get ANC from a health care provider. Only 384 (29.7%) respondents knew that a pregnant woman should first start to attend ANC within three months of pregnancy. About 712 (55.7%) of the women’s partners had a positive attitude towards ANC, and 521 (40.7%) never discussed ANC with their partner. Moreover, less than half (556, 43%) had knowledge about pregnancy-related complications.

### Health promotion activities and decision making in the household

More than half (672, 51.9%) of the women had received education on maternal health. The majority (524, 84.2%) received maternal health education from HEW, followed by mass media (47, 7.6%), health care providers (32, 5.1%), family members (14, 2.3%), community health agents (32, 0.5%) and WDA leaders (2, 0.3%). More than half (727, 56.2%) of the respondents’ homes had been visited at least once by a HEW. More than ninety percent of the women lived in a partner-headed household, followed by a household headed by parents (21, 1.6%) and the respondents themselves (20, 1.5%). More than two thirds (900, 69.6%) of the women stated that decision making at the household level was made jointly with the partner, followed by the respondents (205, 15.8%), the partner (172, 13.3%) and parents (7, 1.3%).

### Antenatal care utilization

About 43.8% of the women attended ANC for their previous pregnancy. Six hundred and ninety three (53.6%, 95% CI: 50.8%, 56.3%) of the women reported to have attended ANC at least once during their most recent pregnancy while only 106 (15.3%) made four or more visits. Of those who had attended at least one ANC, 226 (32.6%, 95% CI: 29.6%, 36.6%) initiated their first visit early (≤3 months of pregnancy). About one fifth (19.9%) of women were accompanied by their partner during the course of attending ANC (Table 3).

**Table 3:** Antenatal care utilization among reproductive-aged women in Kersa district, Eastern Ethiopia, 2017.

Variable	Number	Percentage
**Ever use of ANC for previous pregnancies** (n = 1059)		
Yes	464	43.8
No	595	56.2
**ANC utilization for most recent pregnancy** (n = 1294)		
Yes	693	53.6
No	601	46.4
**Partner accompanying during ANC** (n = 693)		
Yes	138	19.9
No	555	80.1
**ANC provider/s** (n = 693)*		
Doctor/health officer	54	7.8
Nurse/midwife	470	67.8
Health extension worker	235	33.9
Other	14	2.1
**Timing of first ANC initiation** (n = 693)		
≤3 months (Early ANC)	226	32.6
>3 months (Late ANC)	457	67.9
Don’t know	10	1.4
**Frequency of ANC** (n = 693)		
Once	79	11.4
Twice	163	23.5
Three times	345	49.8
Four and more	106	15.3
**Place of ANC attendance (**n = 693)*		
Government hospital	15	2.2
Government health centre	492	71
Government health post	204	29.4
Private hospital/clinics	44	6.3
Home	59	8.5
Other	3	0.4

*Does not sum up to the **total** due to the possibility of multiple responses

**Fig 2:**
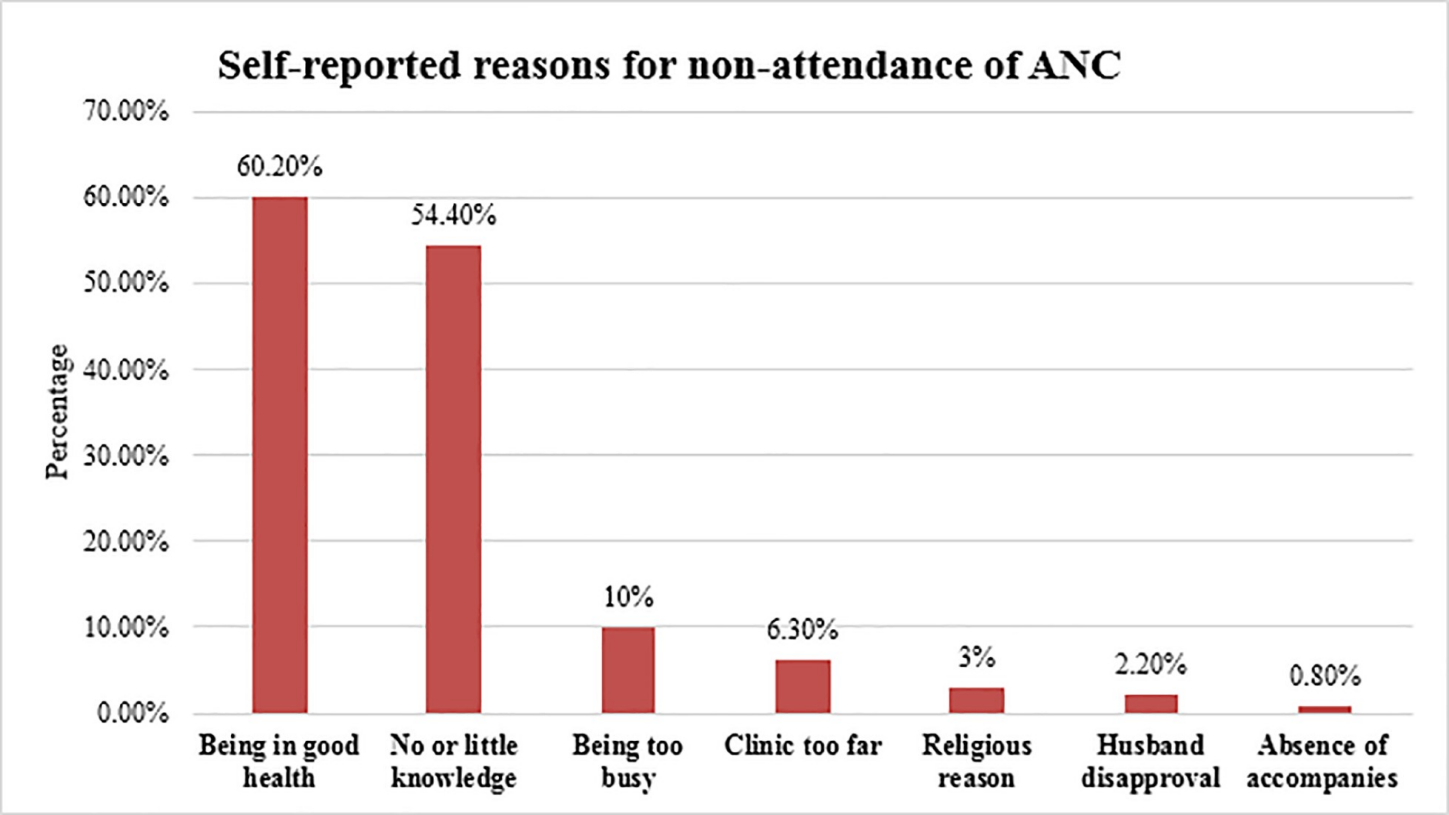
Commonly mentioned self-reported reasons for non-attendance of ANC among reproductive-aged women in Kersa district, Eastern Ethiopia, 2017.

Women had listed their personal reasons for not attending ANC, the most common being that they felt in good health (60.2%), followed by having no or little knowledge (54.4%), and being too busy to attend ANC (10%) (Fig 2).

### Differentials in timing of the first ANC attendance

**Fig 3:**
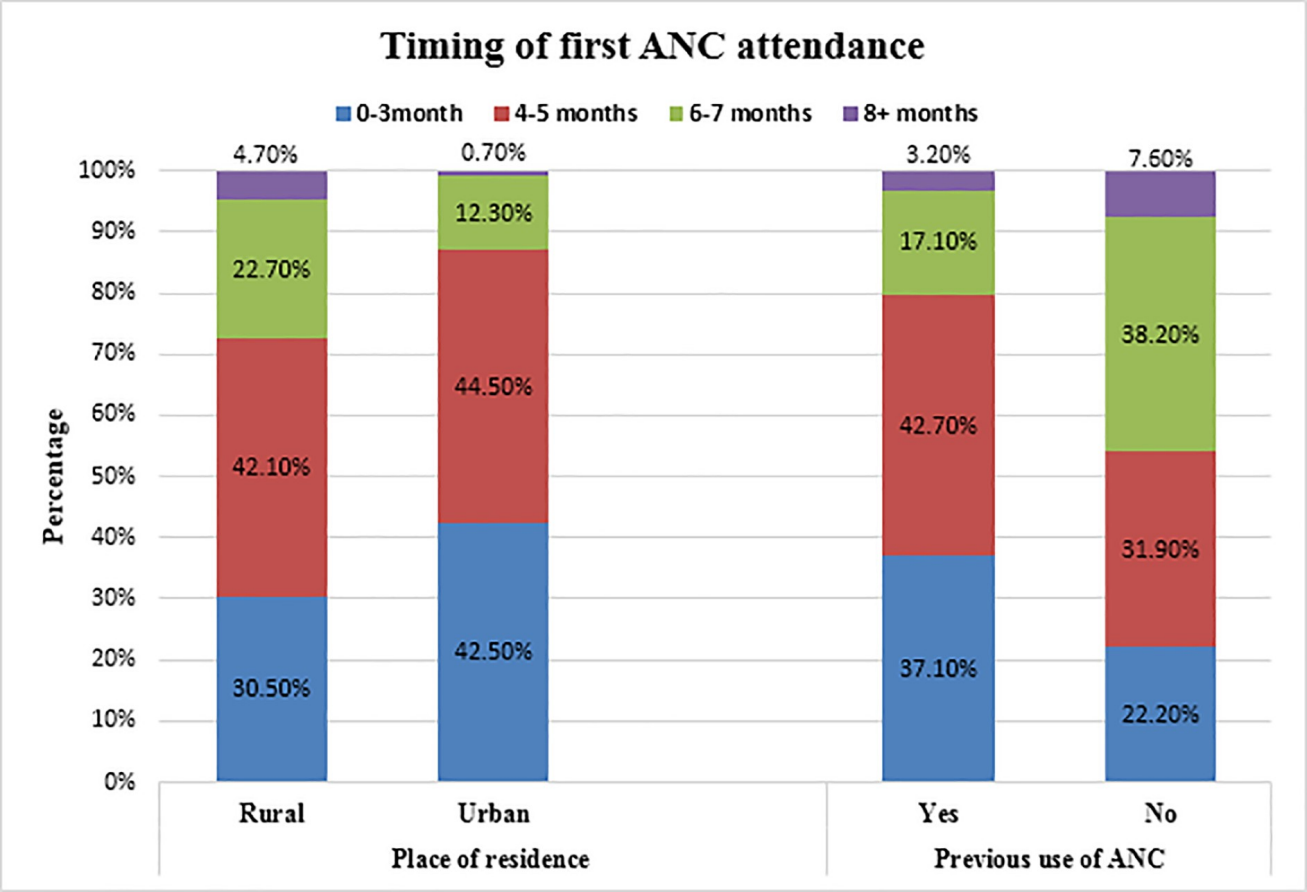
Timing of first ANC by place of residence and previous use of ANC among reproductive-aged women in Kersa district, Eastern Ethiopia, 2017.

A higher proportion of women (42.5%) in urban areas initiated their first ANC visit during the first three months of pregnancy compared with rural women (30.5%). Likewise, significantly more women (37.1%) who previously used ANC presented for the first ANC during the first three months of pregnancy than women who had not used ANC previously (22.2%) (Fig 3).

### Service experience of ANC attendees

Among women who had attended ANC at least once, a large proportion (621, 89.6%) had received tetanus toxoid (TT) injections during their ANC and the majority (87.6%) had received the injection two or more times. A large number (651 or 93.9%) of the women received information about delivery at a health facility from the provider. Blood pressure was measured always in 361 (52.1%) cases, in some visits in 173 (25%) cases, never measured in 144 (20.8%) cases, and 15 women reported that they didn’t know (2.2%). Weight was reported as measured always by 337 (48.6%) respondents, on some visits by 182 (26.3%), never measured in 62 (23.4%), and 12 didn’t know (1.7%). A laboratory examination was never carried out as reported by 330 (47.6%) women, whereas a physical examination was not done for 278 (40.1%) women. Health workers were reported as being respectful by 657 (94.8%) women during the ANC visit and 509 (73.4%) claimed that there was a lack of privacy while receiving ANC. About 440 (63.5%) women were able to ask questions of the provider and 582 (84%) women were reminded by the provider to return again for an ANC visit. Seventy one (10.2%) of the ANC attendees were requested to pay for the service, and only 35 (5.1%) of the women felt the waiting time was a problem, as most (644, 92.9%) of the women received ANC within 2 hr or less. A total of 578 (83.4%) women received ANC from a health facility which was located within 5 km of their home.

### Factors associated with ANC utilization

The results of the multivariate logistic regression analysis are presented in Table 4. We fitted two separate models for the “*ANC at least one*” outcome for multiparous (*m1*) and primiparous women (*m2*). After adjusting for potential confounders in the multivariate logistic regression model of all factors of interest considered in the three domains of ANBM, educational status, previous use of ANC and best friend’s use of maternal care were the predisposing factors that significantly predict an attendance of at least one ANC visit. Primiparous women who had attended formal education were three times more likely to attend at least one ANC visit (AOR, 3.02; 95% CI: 1.26, 7.25) than those who never attended education. Multiparous women who had no previous experience of ANC attendance (AOR, 0.05; 95% CI: 0.03, 0.07) were less likely to attend ANC at least once compared to women who previously attended ANC. Women whose best friends did not utilize maternal care were less likely to attend at least one ANC ((AOR, 0.53; 95% CI: 0.36, 0.78) in *m1* and (AOR, 0.27; 95% CI: 0.12, 0.63) in *m2)* for their most recent pregnancy compared with women whose best friends utilized maternal care.

Enabling factors such as type of *kebele*, wealth index, and husband’s attitude towards ANC showed statistically significant associations with attendance of at least one ANC visit for the most recent pregnancy. Wealth index and husband’s attitude showed a statistically significant association in both models of at least one ANC. Multiparous women who lived in HDSS site had an increased chance of using at least one ANC consultation than those who lived in non-HDSS sites (AOR, 1.60; 95% CI: 1.07, 2.40). Women who belonged to the higher wealth quintiles (*especially in the fourth quintile*) were more likely to attend at least one ANC visit ((AOR, 2.14; 95% CI: 1.22, 3.76) in *m1* and (AOR, 5.90; 95% CI: 1.74, 20.03) in *m2*)) than women in the lower wealth quintiles. Women whose husbands had a negative or neutral attitude towards ANC (AOR, 0.37; 95% CI: 0.25, 0.54) in *m1* and (AOR, 0.27; 95% CI: 0.11, 0.62) in *m2*) were less likely to attend ANC at least once than women whose husbands had a positive attitude. The study also demonstrated that the odds of using four or more ANC check-ups is lower for women whose household was headed by their partner or parents (AOR, 0.16; 95% CI: 0.05, 0.58) compared to women whose household was headed by themselves. Moreover, women whose husbands have a negative or neutral attitude towards ANC have less odds of attending four or more ANC visits (AOR, 0.41; 95% CI: 0.23, 0.75) than women whose husbands have a positive attitude.

Lastly, HEW home visits, perceived importance of ANC and awareness of pregnancy complications were the need factors that showed a significant association with attending at least one ANC visit. However, only the factor “*awareness of pregnancy complication*” showed a significant association in both models. Multiparous women who had never received a HEW home visit (AOR, 0.57; 95% CI: 0.38, 0.84) were less likely to attend at least one ANC visit than their counterparts. Women who had no knowledge of pregnancy complications were less likely to attend at least one ANC compared to women who were aware of pregnancy complications (AOR, 0.52: 95% CI: 0.35, 0.79) in *m1* and (AOR, 0.15; 95% CI: 0.06, 0.40) in *m2*). Multiparous women who perceived that attending ANC is important were more likely to attend at least one ANC visit compared to those who perceived it is not important (AOR, 1.89; 95% CI: 1.17, 3.06). Women who had no awareness of pregnancy complications have less odds of attending four or more ANC visits compared to women who have awareness (AOR, 0.51; 95% CI: 0.30, 0.85). Women who had an abortion history were more likely to attend four or more ANC visits than their counterparts (AOR, 2.06; 95% CI: 1.05, 4.04).

**Table 4:** Factors associated with ANC utilization among reproductive aged women in Kersa district, Eastern Ethiopia, 2017.

*Predisposing factors*	*Categories*	ANC at least one AOR (95% CI) (*n = 1059*) *m1*	ANC at least one AOR (95% CI) (*n = 235*) *m2*	ANC 4+ AOR (95% CI) (*n = 693*)
**Educational status**	Never attended	1	1	1
	Attended	1.50(0.92, 2.46)	3.02(1.26, 7.25)*	1.18(0.69, 2.02)
**Husband’s education**	Never attended	1	1	1
	Attended	1.21(0.83, 1.74)	1.91(0.81, 4.48)	1.06(0.62, 1.79)
**Education on maternal health**	Yes	0.96(0.66, 1.40)	1.71(0.74, 3.97)	
	No	1	1	
**Mass media availability**	Yes	1.01(0.66, 1.52)	1.10(0.45, 2.62)	
	No	1	1	
**Telephone (mobile) ownership**	Yes	0.73(0.45, 1.17)	1.03(0.39, 2.77)	
	No	1	1	
**Age at first marriage**	*Continuous*			1.09(0.92, 1.28)
**Age at first pregnancy**	*Continuous*			1.03(0.88, 1.21)
**Birth order**	≤3^rd^	1		
	>3^rd^	0.85(0.59, 1.25)		
**Previous use of ANC**	Yes	1		
	No	0.05(0.03, 0.07)*		
**Living in a model family**	Yes	1		1
	No	1.15(0.31, 4.24)		0.58(0.25, 1.35)
**Best friend’s use of maternal care**	Yes	1	1	
	No/don’t know	0.53(0.36, 0.78)**	0.27(0.12, 0.63)**	
**Parity**	Yes			1
	No			0.73(0.43, 1.25)
***Enabling factors***				
**Residence**	Rural	1	1	1
	Urban	0.50(0.22, 1.14)	0.94(0.21, 4.15)	1.07(0.58, 1.96)
**Type of *kebele***	HDSS	1.60(1.07, 2.40)*	1.61(0.66, 3.92)	
	Non-HDSS	1	1	
**Wealth index**	Highest	1.52(0.85, 2.70)	6.87(1.81, 26.01)*	
	Fourth	2.14(1.22, 3.76)**	5.90(1.74, 20.03)**	
	Middle	1.21(0.69, 2.12)	5.46(1.57, 18.95)*	
	Second	1.68(0.98, 2.89)	3.97(1.12, 13.44)*	
	Lowest	1	1	
**Decision making on household expenses**	Respondent	1	1	
	Jointly	1.50(0.91, 2.48)	1.99(0.64, 6.20)	
	Partner/parents	1.34(0.66, 2.71)	2.07(0.50, 8.03)	
**Head of the household**	Respondent			1
	Partner/parents			**0.16(0.05, 0.58)**
**Husband’s attitude towards ANC**	Positive	1	1	1
	Negative/neutral	0.37(0.25, 0.54)**	0.27(0.11, 0.62)**	**0.41(0.23, 0.75)**
**Social support from friends**	Yes	1	1	
	No	0.69(0.37, 1.29)	0.67 (0.24, 1.83)	
***Need factors***				
**HEW home visit**	Yes	**1**		
	No	0.57(0.38, 0.84)*		
**Pregnancy intention**	Intended	1		
	Unintended	0.73(0.49, 1.09)		
**History of abortion**	Has no history			1
	Has history			**2.06(1.05, 4.04)**
**Awareness of pregnancy complications**	Yes	1	1	1
	No	0.52(0.35, 0.79)**	0.15(0.06, 0.40)**	**0.51(0.30, 0.85)**
**Perceived importance of ANC attendance**	Not important	1	1	
	Important	1.89(1.17, 3.06)*	2.12(0.74, 6.08)	

**Key: *AOR***: Adjusted Odds Ratio (adjusting for all the ***predisposing***, ***enabling*** and ***need*** factors in final full model), ***CI***: Confidence Interval (95%)

Bold*: statistically significant variables

** statistically significant variables in the two models (*m1 and m2*) of “*at least one ANC*” outcome

## Discussion

In this study, we set out to examine the factors associated with ANC utilization in Kersa district, Eastern Ethiopia. We found that women’s education, previous use of ANC and best friend’s use of maternal care were significant predisposing factors associated with at least one ANC attendance. Furthermore, type of *kebele*, wealth index and husband’s attitude towards ANC were significant enabling factors influencing at least one ANC use. HEW household visits, perceived importance of ANC attendance, and awareness of pregnancy complications were significant need factors that predicted at least one ANC attendance. Being head of the household, having abortion history, having awareness about pregnancy complications and husband’s attitude towards ANC were factors that predicted an attendance of four or more ANC visits.

In the study, it was demonstrated that 53.6% of the women received at least one ANC check-up for the index pregnancy. This finding is consistent with a study conducted in Tigray, Northern Ethiopia [24], where 54% of the women received ANC at least once for their last pregnancy. The finding is also comparable with the Oromia region average (51%) where the current study district is located. However, the level of ANC uptake observed in this study is lower than that of the national average (62%) [19] and the findings from many other studies in different regions of Ethiopia [13, 14, 16, 18]. The exhibited disparities in the magnitude of ANC utilization might be attributed to the inter-regional difference in geographical area, socioeconomic and cultural settings in Ethiopia. For instance, in some regions of Ethiopia, there is an underdeveloped health care system whereas in others the people live in nomadic areas where health service is rarely available. Moreover, each region in Ethiopia has its own peculiar socio-economic features and cultural practices that can either positively or negatively influence health care utilization patterns. The current study was conducted in a largely rural area of the country where there are high socio-cultural barriers and poor community perceptions towards utilization of ANC [36] that would probably have reduced ANC uptake.

Furthermore, in this study, the proportion of women who attended four or more ANC visits was only 15.3%. This result is lower than in a study conducted in Kombolcha district, Eastern Ethiopia (38.3%) [18] but higher than in a study conducted in Tigray, Northern Ethiopia (6.4%) [17]. The differences may be related to the fact that the studies were conducted at different points in time and the level of safe motherhood primary health care activities might be different in various parts of the country. It is recommended that further studies be conducted at some period after the new WHO guideline (having a minimum of eight ANC visits) [1] is implemented in Ethiopia. Antenatal care is more effective in averting unfavourable pregnancy complications if given early in the pregnancy [21]. However, the current study showed that only one third (32.6%) of the women initiated ANC before the 12^th^ week of gestation. The result is in line with a systematic review finding [22], where only 36% of pregnant women in Ethiopia started to attend ANC during the early stages of pregnancy.

The result of the multivariate analysis indicated that women’s utilization of ANC is affected by predisposing, enabling and need factors. Predisposing factors including women’s education, previous use of ANC and women’s best friend’s use of maternal care were predictors of ANC utilization. Women might be informed about the benefits of ANC utilization in various ways, such as exposure to mass media, information exchange with friends, community-based health education and previous exposure to health services. The current study provided evidence that shows multiparous women who had obtained ANC services for their previous pregnancy were more likely to attend at least one ANC consultation for subsequent pregnancies. It has been previously documented [5, 37] that obtaining maternal care in the previous pregnancy positively influenced the utilization of the service for the subsequent pregnancy. This might be related to the fact that women could be better informed about the benefit of ANC through the previous service attendance.

Women’s education showed an association with ANC utilization, particularly among primiparous women. Demographically, primiparous women (*those who only had one delivery*) are often younger and more educated than multiparous women, which might make this variable more significantly predict ANC utilization for this group of women than multiparous women. Evidence suggests that younger women [16, 17, 38] and those with fewer deliveries [16, 38] have increased odds of ANC utilization. Education is a very important factor that influences behaviour and the changing attitudes of individuals [39]. Women’s education helps not only to increase their empowerment but also transform their social value, which enables women to have improved access to maternal care and enhances their ability to utilize it. The positive influence of education on ANC utilization is well documented in several prior studies in Ethiopia [13, 16, 17, 25].

Best friend’s use of maternal care is another predisposing factor associated with ANC utilization in this study. Women whose best friends use maternal care were more likely to attend at least one ANC visit compared to women whose best friends did not use maternal care. It has been illustrated in the systematic review [40] of ANC utilization among non-western women that acquiring and following advice from friends or peers has facilitated better ANC utilization for pregnant women. A study in India [41], also reported that a woman’s social network had a positive correlation with ANC utilization. Social networks significantly influence the decision-making and health-seeking behaviour of pregnant women towards ANC, which ultimately facilitates or hinders their access to and utilization of the service [27]. The social ties that individuals develop, whether their families, friends or peer groups are essential to spanning interactions with better informed opinion leaders inside the community who could, in turn, sway the people’s behaviour or actions towards the use of preventive health care services [41–44]. Results that demonstrated the positive influence of best friends highlights a largely untapped resource for public health messaging in Ethiopia. Maternal health promotion activities should involve spreading messages regarding the benefit of attending ANC for the health of the mother and the child by targeting the social networks of women through peer education programs.

Enabling factors such as type of *kebele*, wealth index, and husband’s attitude were independently associated with ANC utilization. Women in the fourth wealth index category were more likely to use ANC compared to women in other lower wealth categories. This was consistent with a study conducted in Southern Ethiopia [13], where women who belonged to the higher wealth quintiles had higher odds of ANC attendance that women in lower quintiles. This could be due to the fact that women with higher economic status could easily afford to cover health care and transportation costs. Moreover, women with high economic status tend to be more educated and this would, in turn, increase their service use. Therefore, economic reform activities in Ethiopia should involve women and poorer families to subsequently increase women’s ANC uptake.

Multiparous women who live in the HDSS site were more likely to use ANC services than those who live out of the HDSS site. This was in line with a study conducted in southern Ethiopia [13] in which living in a HDSS site is to some extent protective of maternal death resulting from non-use of services. It is expected that demographic and health surveillance sites are exposed to continuous surveys and health information as there could be regular visits by field enumerators and researchers. Evidence [45] suggests that populations under HDSS have better health indicators compared to populations not under surveillance because of repeated data collection and measurements which could function as a passive intervention resulting in behaviour change.

A husband’s attitude towards ANC also influenced women’s utilization of ANC in this study. The risk of non-attendance of at least one ANC was high for women whose husband’s attitude was negative or neutral. The effect of a husband’s attitude on women’s utilization of ANC has been well documented in other studies conducted in different parts of Ethiopia [12, 46]. A husband’s attitude towards ANC influences their tendency to approve the care to their wives. As most women in developing countries need a husband’s approval to seek health services, including ANC, a husband should have a supportive attitude towards health services, thereby allowing his wife to attend the services. Hence, intervention efforts should be in place to improve husband’s attitudes towards ANC to increase the uptake of the service. One such intervention may be designing messages about maternal health and diffusing this through community-based men’s social networks to improve their knowledge. Also, as more educated men tend to be more involved in their spouse care [47], improving men’s educational status is essential.

HEW home visits, perceived importance of ANC attendance and awareness of complications that occur during pregnancy are significant predictors of at least one ANC consultation. Women who received a HEW home visit were more likely to attend at least one ANC visit compared to women who never received a HEW visit. A similar study finding was reported from a study conducted in southern Ethiopia [48]. Access to HEW home visits should be further strengthened to reach women while they are at their home to effectively provide health messages and promote ANC utilization. Increasing the frequency of contact and improving the continuity of HEW home visits through expanding the HEW’s outreach service operation hours is crucial, particularly for reaching pregnant women in underserved communities. Moreover, upgrading the capacity of the HEWs to provide the full package of home-based ANC services apart from the routine health education and promotion activity is required.

Perceived importance of ANC attendance showed a positive association with ANC utilization, where multiparous women who believe that attending the service is important were more likely to attend the care. The finding of the study was in accordance with a study conducted in Lao PDR [49] where women who had a positive attitude towards the benefits of ANC service were more likely to use these services than those with a negative attitude about the benefit of the service. Moreover, in a different study in Ethiopia, it was demonstrated that knowledge of the benefits of antenatal care services was significantly associated with women’s utilization of early ANC services [50]. The finding highlights the need to establish strategies that aim to improve women’s awareness of the importance of ANC attendance using community-based education programs, especially among multiparous women.

The current study also revealed that women who have an awareness of pregnancy complications were more likely to attend ANC than their counterparts. The results of this study were comparable with other studies in Ethiopia [14–16] where the odds of ANC utilization were higher among women with a better awareness of pregnancy complications than those with poor knowledge. The result highlights the need to design intervention strategies that involve promoting awareness about pregnancy-related complications among women.

### Modifiable factors for program or policy consideration

**Fig 4:**
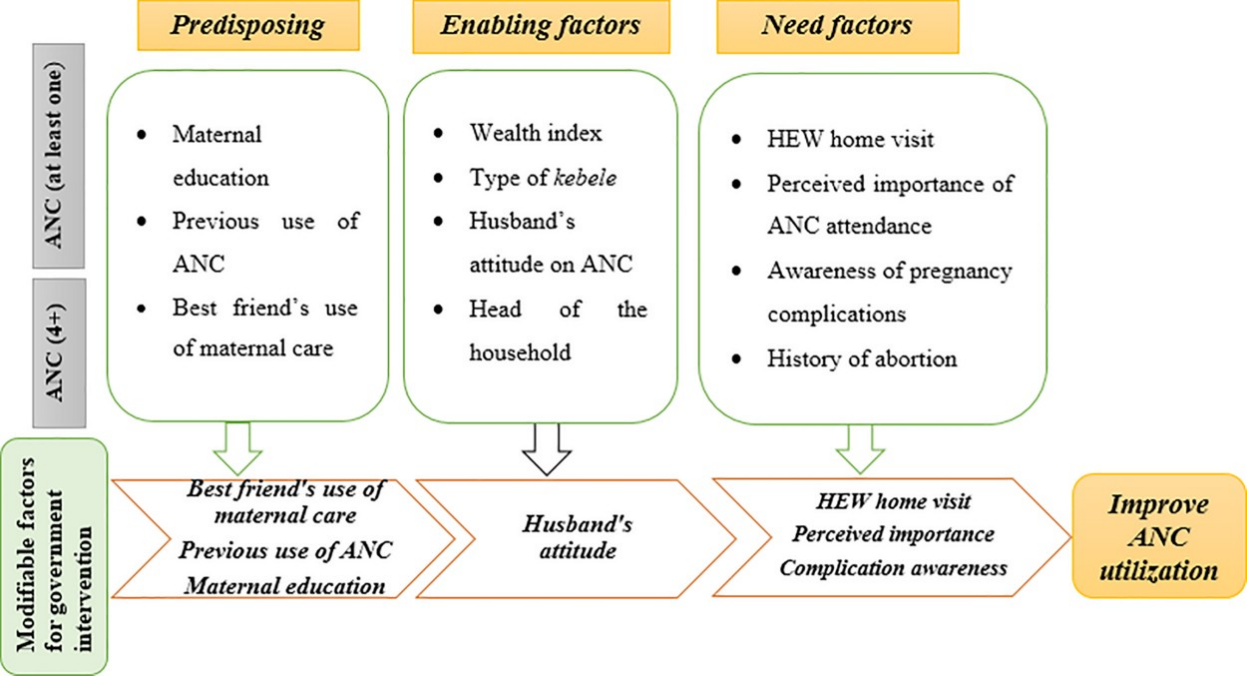
Modifiable predisposing, enabling and need factors associated with ANC utilization for government intervention based on ANBM, 2017.

The factors that are amenable to modification in the context of Ethiopia for further government and stakeholders’ interventions to increase ANC uptake are illustrated below. Predisposing factors that can be targeted by the government to mitigate poor utilization of ANC include women’s education, previous use of ANC and best friend’s use of maternal care. Enabling factors such as husband’s attitude towards ANC can also be targeted to increase ANC attendance. Lastly, increasing HEW home visits, increasing women knowledge on the importance of ANC attendance, and promoting awareness of obstetric complications could be potential areas of intervention to further improve ANC utilization in Ethiopia (Fig 4).

### Strengths and limitations of the study

We believe that inclusion of data regarding only the most recent pregnancy that occurred within the three years preceding the survey could minimize the risk of recall bias. We considered many predictor variables from the three domains of ANBM into one model in order to better control for potential confounders. Use of iPads with digital survey tools for the data collection is another strength of the study because missing data are minimized. One limitation of the study was reliance on self-reporting of data. In addition, due to the cross-sectional nature of the study, it is difficult to establish a temporal relationship between the predictors and outcome variables. Though training was provided to the interviewers to inform respondents about the purpose of the study, the study might still be prone to social desirability bias. Despite these limitations, the findings of the study are sufficiently valid to inform program development.

## Conclusion

More than half of the women attended ANC at least once during their last pregnancy. A higher proportion of women attended less than four ANC check-ups and delayed their first ANC visit. Attending formal education, belonging to higher wealth quintiles, previous use of ANC, best friend’s use of maternal care, living in HDSS site, husband’s positive attitudes towards ANC, receiving HEW home visits, perceiving ANC attendance as important, and having an awareness of pregnancy complications were drivers for attendance of at least one ANC. Moreover, partner or parents being head of household, not experiencing an abortion, poor awareness of pregnancy complications, and husband’s negative or neutral attitude towards ANC were deterrents for attending four or more ANC visits. Maternal health intervention efforts in Ethiopia should involve strategies that focus on the following key areas: improving women’s education enrolment, designing women’s peer education programs to mobilize and support women, programs to change husband’s attitude through community mobilization, women-centred economic reforms, ameliorating service provision of ANC, strengthening the HEW home visits programs to further expand the health promotion and diseases prevention activities, and designing programs to increase awareness about pregnancy complications and importance of practicing ANC during pregnancy, especially for underprivileged women in rural areas.

## Supporting information

S1 Table Model building process to identify potential factors associated with at least one antenatal care visit among reproductive aged women in Kersa district, Eastern Ethiopia, 2017. (DOCX)

S2 Table Model building process to identify factors associated with four or more antenatal care attendance among reproductive aged women in Kersa district, Eastern Ethiopia, 2017. (DOCX)

S1 File Data collection tool for ANC section (English). (DOCX)

S2 File Data collection tool for ANC section (Local language). (DOCX)
