# Supplementary material for: Application of the Andersen-Newman model of health care utilization to understand antenatal care use in Kersa District, Eastern Ethiopia
Source: PLoS One. 2018 Dec 6;13(12):e0208729. doi: 10.1371/journal.pone.0208729 (PMC6283597; doi:10.1371/journal.pone.0208729)
Supplement: S1 Table — (DOCX) [file pone.0208729.s001.docx]

**Table 1**: Model building process to identify potential factors associated with at least one antenatal care visit among reproductive aged women in Kersa district, Eastern Ethiopia, 2017 (n=1059, multiparous women)

| ***Predisposing factors*** | ***Categories*** | **ANC** (at least one visit) | | **COR (95% CI)** | **Model 1 (AOR at 95% CI)** | **Model 2 (AOR at 95% CI)** | **Model 3 (AOR at 95% CI)** |
| --- | --- | --- | --- | --- | --- | --- | --- |
|  |  | **No (%)** | **Yes (%)** |  |  |  |  |
| **Maternal age** | *Continuous* | 498(47%) | 561(53%) | 0.99(0.97,1.02) | *Excluded* | *Excluded* | *Excluded* |
| **Educational status** | Never attended | 431(52.7%) | 383 (47.1%) | 1 | 1 | 1 | 1 |
|  | Attended | 67 (27.3%) | 178 (72.7%) | 2.99 (2.19,4.09) | 1.47(0.93,2.32) | 1.53(0.94,2.48) | 1.50(0.92, 2.46) |
| **Husband’s education** | Never attended | 316 (55.4%) | 254 (44.6%) | 1 | 1 | 1 | 1 |
|  | Attended | 182 (37.2%) | 307 (62.8%) | 2.10 (1.64,2.69) | 1.34(0.95,1.90) | 1.25(0.87,1.80) | 1.21(0.83, 1.74) |
| **Education on maternal health** | Yes | 201 (39.0%) | 315 (61%) | 1.89 (1.48,2.42) | 1.33(0.95,1.85) | 1.16(0.81,1.64) | 0.96(0.66,1.40) |
|  | No | 297 (54.7%) | 246 (45.3%) | 1 | 1 | 1 | 1 |
| **Mass media availability** | Yes | 126 (36.2%) | 339 (63.8%) | 1.93(1.49, 2.52) | 1.01(0.69,1.49) | 1.01(0.66,1.52) | 1.01(0.66,1.52) |
|  | No | 372 (52.3%) | 397 (47.7%) | 1 | 1 | 1 | 1 |
| **Telephone (mobile) ownership** | Yes | 90 (34.4%) | 172 (65.6%) | 2.01 (1.50,2.68) | 0.84(0.55,1.30) | 0.75(0.47,1.19) | 0.73(0.45, 1.17) |
|  | No | 408 (51.2%) | 389 (48.8%) | 1 | 1 | 1 | 1 |
| **Age at first marriage** | *Continuous* | 498(47%) | 561(53%) | 1.03(0.98,1.09) | *Excluded* | *Excluded* | *Excluded* |
| **Age at first pregnancy** | *Continuous* | 498(47%) | 561(53%) | 1.04(0.99,1.09) | *Excluded* | *Excluded* | *Excluded* |
| **Birth order** | ≤3^rd^ | 188 (43.1%) | 248 (56.9%) | 1 | 1 | 1 | 1 |
|  | >3^rd^ | 310 (49.8%) | 313 (50.2%) | 0.77(0.60,0.98) | 0.98(0.70,1.39) | 0.99(0.69,1.43) | 0.85(0.59, 1.25) |
| **Previous use of ANC** | Yes | 49 (10.5%) | 417 (89.5%) | 1 | 1 | 1 | 1 |
|  | No | 449 (75.7%) | 144 (24.3%) | 0.04(0.03,0.05) | 0.05(0.04,0.07) | 0.05(0.03,0.07) | **0.05(0.03, 0.07)** |
| **Living in model family** | Yes | 6(15.2%) | 28 (84.8%) | 1 | 1 | 1 | 1 |
|  | No | 493 (48.1%) | 533 (51.9%) | 0.19(0.07,0.50) | 0.84(0.25,2.85) | 0.99(0.28,3.52) | 1.15(0.31, 4.24) |
| **Best friend’s use of care** | Yes | 110 (24.8%) | 334 (75.2%) | 1 | 1 | 1 | 1 |
|  | No/don’t know | 388 (63.1%) | 227 (37.9%) | 0.19(0.15,0.25) | 0.37(0.26,0.52) | 0.46(0.32,0.67) | **0.53(0.36, 0.78)** |
| ***Enabling factors*** |  |  |  |  |  |  |  |
| **Residence** | Rural | 475 (50.7%) | 462 (49.3%) | 1 |  | 1 | 1 |
|  | Urban | 23 (18.9%) | 99 (81.1%) | 4.43(2.76,7.09) |  | 0.62(0.29,1.35) | 0.50(0.22, 1.14) |
| **Type of kebele** | HDSS | 298 (42.0%) | 411 (58.0%) | 1.84(1.42,2.38) |  | 1.65(1.13,2.43) | **1.60(1.07, 2.40)** |
|  | Non-HDSS | 200 (57.1%) | 150 (42.9%) | 1 |  | 1 | 1 |
| **Distance from nearest health facility** | ≥5Km | 13 (36.1%) | 23 (63.9%) | 1 |  |  |  |
|  | <5Km | 485 (47.4%) | 538 (52.6%) | 0.63(0.31,1.25) |  | *Excluded* | *Excluded* |
| **Wealth index** | Highest | 92 (44.9%) | 113 (55.1%) | 1.22(0.83,1.79) |  | 1.34(0.76,2.37) | 1.52(0.85, 2.70) |
|  | Fourth | 82 (39.4%) | 126 (60.6%) | 1.52(1.03,2.24) |  | 1.96(1.12,3.42) | **2.14(1.22, 3.76)** |
|  | Middle | 119 (55.3%) | 96 (44.7%) | 0.80(0.55,1.17) |  | 1.15(0.66,2.01) | 1.21(0.69, 2.12) |
|  | Second | 101 (45.5%) | 121 (54.5%) | 1.19(0.81,1.73) |  | 1.57(0.91,2.68) | 1.68(0.98, 2.89) |
|  | Lowest | 104 (49.8%) | 105 (50.2%) | 1 |  | 1 | 1 |
| **Decision making on household expenses** | Respondent | 91 (52.0%) | 84 (48.0%) | 1 |  | 1 | 1 |
|  | Jointly | 336 (45.1%) | 409 (54.9%) | 1.32(0.95,1.83) |  | 1.58(0.96,2.58) | 1.50(0.91, 2.48) |
|  | Partner/parents | 71 (51.1%) | 68 (48.9%) | 1.04(0.67,1.62) |  | 1.27(0.65,2.51) | 1.34(0.66, 2.71) |
| **Head of the household** | Respondent | 6 (35.3%) | 11 (64.7%) | 1 |  |  |  |
|  | Partner/parents | 492 (47.2%) | 550 (52.8%) | 0.61(0.22,1.66) |  | *Excluded* | *Excluded* |
| **Husband’s attitude towards ANC** | Positive | 179 (31.3%) | 393 (68.7%) | 1 |  | 1 | 1 |
|  | Negative/neutral | 319 (65.5%) | 168 (34.5%) | 0.24(0.19,0.31) |  | 0.33(0.23,0.47) | **0.37(0.25, 0.54)** |
| **Social support from friends** | Yes | 433 (45.2%) | 525 (54.8%) | 1 |  | 1 | 1 |
|  | No | 65 (64.4%) | 36 (35.6%) | 0.46(0.30,0.70) |  | 0.66(0.35,1.23) | 0.69(0.37, 1.29) |
| ***Need factors*** |  |  |  |  |  |  |  |
| **HEW home visit** | Yes | 237 (38.6%) | 377 (61.4%) | 1 |  |  | **1** |
|  | No | 261 (58.7%) | 184 (41.3%) | 0.44(0.35,0.57) |  |  | **0.57(0.38, 0.84)** |
| **Pregnancy intention** | Intended | 299 (42.1%) | 411 (57.9%) | 1 |  |  | 1 |
|  | Unintended | 199 (57.0%) | 150 (43.0%) | 0.55(0.42,0.71) |  |  | 0.73(0.49, 1.09) |
| **History of abortion** | Has no history | 465 (47.7%) | 509 (52.3%) | 1 |  |  |  |
|  | Has history | 33 (38.8%) | 52 (61.2%) | 1.44 (0.91,2.27) |  |  | *Excluded* |
| **Awareness of pregnancy complications** | Yes | 183 (39.2%) | 284 (60.8%) | 1 |  |  | 1 |
|  | No | 315 (53.2%) | 277 (46.8%) | 0.57(0.44,0.73) |  |  | **0.52(0.35, 0.79)** |
| **History of still birth** | Has no history | 451 (46.7%) | 514 (53.3%) | 1 |  |  |  |
|  | Has history | 47 (50.0%) | 47 (50.0%) | 0.88(0.57,1.34) |  |  | *Excluded* |
| **History of infant death** | Has no history | 368 (46.2%) | 428 (53.8%) | 1 |  |  |  |
|  | Has history | 130 (49.4%) | 133 (50.6%) | 0.88(0.66,1.16) |  |  | *Excluded* |
| **Perceived importance of ANC** | Not important | 179(72.8%) | 67(27.2%) | 1 |  |  | 1 |
|  | Important | 319(39.2%) | 494(40.8%) | 4.14(3.02,5.66) |  |  | **1.89 (1.17, 3.06)** |

**Keys**: **Model 1** (Predisposing factors only), **Model 2** (Predisposing and enabling factors), **Model 3** (Predisposing, enabling and need factors). **AOR**: Adjusted Odds Ratios, **COR:** Crude Odds Ratios, **CI:** Confidence Interval. **Bold**: Indicate statistically significant variables.
