# Supplementary material for: Application of the Andersen-Newman model of health care utilization to understand antenatal care use in Kersa District, Eastern Ethiopia
Source: PLoS One. 2018 Dec 6;13(12):e0208729. doi: 10.1371/journal.pone.0208729 (PMC6283597; doi:10.1371/journal.pone.0208729)
Supplement: S2 Table — (DOCX) [file pone.0208729.s002.docx]

**Table 1**: Model building process to identify factors associated with four or more antenatal care attendance among reproductive aged women in Kersa district, Eastern Ethiopia, 2017 (n=693)

| ***Predisposing factors*** | ***Categories*** | **ANC 4+** | | **COR (95% CI)** | **Model 1 (AOR with 95% CI)** | **Model 1 (AOR with 95% CI)** | **Model 1 (AOR with 95% CI)** |
| --- | --- | --- | --- | --- | --- | --- | --- |
|  |  | **No** | **Yes** |  |  |  |  |
| **Maternal age** | *Continuous* | 587 (84.7%) | 106 (15.3%) | 0.98(0.95,1.02) | *Excluded* | *Excluded* | *Excluded* |
| **Educational status** | Never attended | 374 (87.8%) | 52 (12.2%) | 1 |  | 1 | 1 |
|  | Attended | 213 (79.8%) | 54 (20.2%) | 1,82 (1.20,2.77) | 1.36(0.83,2.21) | 1.13 (0.67,1.92) | 1.18(0.69,2.02) |
| **Husband’s education** | Never attended | 250 (88.7%) | 32 (11.3%) | 1 |  | 1 | 1 |
|  | Attended | 337 (82.0%) | 74 (12.0%) | 1.72 (1.10,2.68) | 1.30(0.79,2.16) | 1.16(0.69,1.95) | 1.06(0.62,1.79) |
| **Education on maternal health** | Yes | 332 (85.8%) | 55 (14.2%) | 0.83 (0.55,1.25) | *Excluded* | *Excluded* | *Excluded* |
|  | No | 255 (83.3%) | 51 (16.7%) | 1 |  |  |  |
| **Mass media availability** | Yes | 245 (82.8%) | 51 (17.2%) | 1.29 (0.85,1.96) | *Excluded* | *Excluded* | *Excluded* |
|  | No | 342 (86.1%) | 55 (13.9%) | 1 |  |  |  |
| **Telephone (mobile) ownership** | Yes | 193 (81.1%) | 45 (18.9%) | 1.51 (0.98, 2.30) | *Excluded* | *Excluded* | *Excluded* |
|  | No | 394 (86.6%) | 61 (13.4%) | 1 |  |  |  |
| **Age at first marriage** | *Continuous* | 587 (84.7%) | 106 (15.3%) | 1.15(1.06,1.25) | 1.06(0.90,1.24) | 1.08(0.92,1.27) | 1.09(0.92,1.28) |
| **Age at first pregnancy** | *Continuous* | 587 (84.7%) | 106 (15.3%) | 1.15(1.06,1.25) | 1.06 (0.91,1.25) | 1.02(0.87,1.19) | 1.03(0.88,1.21) |
| **Previous use of ANC** | Yes | 356 (85.4%) | 61 (14.6%) | 1 |  |  |  |
|  | No | 128 (88.3%) | 17 (11.7%) | 0.77(0.44,1.38) | *Excluded* | *Excluded* | *Excluded* |
| **Birth order** | ≤3^rd^ | 314 (82.6%) | 66 (17.4%) | 1 |  |  | 1 |
|  | >3^rd^ | 273 (87.2%) | 40 (12.8%) | 0.70 (0.46,1.07 | *Excluded* | *Excluded* | *Excluded* |
| **Living in model family** | Yes | 23 (71.9%) | 9 (28.1%) | 1 | 1 | 1 | 1 |
|  | No | 564 (85.3%) | 97 (14.7%) | 0.44(0.20,0.98) | 0.49 (0.21,1.14) | 0.53(0.23,1.22) | 0.58(0.25,1.35) |
| **Best friend’s use of care** | Yes | 350 (83.9%) | 67 (16.1%) | 1 |  |  | 1 |
|  | No/don’t know | 237 (85.9%) | 39 (14.1%) | 0.86(0.56,1.32) | *Excluded* | *Excluded* | *Excluded* |
| **Parity** | Primipara | 104 (78.8%) | 28 (21.2%) | 1 | 1 | 1 | 1 |
|  | Multipara | 483 (86.1%) | 78 (13.9%) | 0.60(0.37,0.97) | 0.77(0.46,1.30) | 0.77(0.46,1.31) | 0.73(0.43, 1.25) |
| ***Enabling factors*** |  |  |  |  |  |  |  |
| **Residence** | Rural | 478 (87.4%) | 69 (12.6%) | 1 |  | 1 | 1 |
|  | Urban | 109 (74.7%) | 37 (25.3%) | 2.35 (1.59,3.69) |  | 1.56(0.91,2.67) | 1.07(0.58,1.96) |
| **Type of kebele** | HDSS | 436 (85.0%) | 77 (15.0%) | 0.92 (0.58,1.47) |  | *Excluded* | *Excluded* |
|  | Non-HDSS | 151 (83.9%) | 29 (14.1%) | 1 |  |  |  |
| **Distance from nearest health facility** | ≥5Km | 22 (84.1%) | 4 (15.9%) | 1 |  |  |  |
|  | <5Km | 565 (84.7%) | 102 (15.3%) | 0.99 (0.34,2.94) |  | *Excluded* | *Excluded* |
| **Wealth index** | Highest | 125 (83.9%) | 24 (16.1%) | 0.84 (0.45,1.58) |  | *Excluded* | *Excluded* |
|  | Fourth | 132 (83.5%) | 26 (16.5%) | 0.87 (0.47,1.61) |  | *Excluded* | *Excluded* |
|  | Middle | 105 (87.5%) | 15 (12.5%) | 0.63 (0.31,1.27) |  | *Excluded* | *Excluded* |
|  | Second | 124 (87.3%) | 18 (12.7%) | 0.64 (0.33,1.25) |  | *Excluded* | *Excluded* |
|  | Lowest | 101 (81.5%) | 23 (18.5%) | 1 |  |  |  |
| **Decision making on household expenses** | Respondent | 83 (85.6%) | 14 (14.4%) | 1 |  |  |  |
|  | Jointly | 436 (86.0%) | 71 (14.0%) | 0.97 (0.52,1.79) |  | *Excluded* | *Excluded* |
|  | Partner/parents | 68 (76.4%) | 21 (23.6%) | 1.83 (0.87,3.87) |  | *Excluded* | *Excluded* |
| **Head of the household** | Respondent | 7 (58.3%) | 5 (41.7%) | 1 |  | 1 | 1 |
|  | Partner/parents | 580 (85.2%) | 101 (14.8%) | 0.24(0.08,0.78) |  | 0.16(0.05,0.56) | **0.16(0.05,0.58)** |
| **Husband’s attitude towards ANC** | Positive | 407 (82.1%) | 89 (17.9%) | 1 |  | 1 | 1 |
|  | Negative/neutral | 180 (91.4%) | 17 (8.6%) | 0.43(0.25,0.75) |  | 0.48(0.27,0.86) | **0.41(0.23,0.75)** |
| **Social support from friends** | Yes | 541 (84.1%) | 102 (15.9%) | 1 |  | 1 | 1 |
|  | No | 46 (92.0%) | 4 (8.0%) | 0.46(0.16,1.31) |  | *Excluded* | *Excluded* |
| ***Need factors*** |  |  |  |  |  |  |  |
| **Pregnancy intention** | Intended | 445 (83.8%) | 86 (16.2%) | 1 |  |  | 1 |
|  | Unintended | 142 (87.7%) | 20 (12.3%) | 0.73 (0.43,1.23) |  |  | Excluded |
| **History of abortion** | Has no history | 543 (85.6%) | 91 (14.4%) | 1 |  |  | 1 |
|  | Has history | 44 (74.6%) | 15 (15.4%) | 2.03 (1.10,3.81) |  |  | **2.06(1.05,4.04)** |
| **Awareness of pregnancy complications** | Yes | 286 (79.9%) | 72 (20.1%) | 1 |  |  | 1 |
|  | No | 301 (89.9%) | 34 (10.1%) | 0.45 (0.29,0.70) |  |  | **0.51(0.30,0.85)** |
| **History of still birth** | Has no history | 544 (84.3%) | 101 (15.7%) | 1 |  |  |  |
|  | Has history | 43 (89.6%) | 5 (10.4%) | 0.63 (0.24,1.62) |  |  | *Excluded* |
| **History of infant death** | Has no history | 470 (84.1%) | 89 (15.9%) | 1 |  |  |  |
|  | Has history | 117 (87.3%) | 17 (12.7%) | 0.77 (0.44,1.34) |  |  | *Excluded* |

**Key*: AOR****:* Adjusted Odds Ratio (adjusting for all the ***predisposing***, ***enabling*** and ***need*** factors in final model), ***CI****:* Confidence Interval (95%), **Bold*:*** statistically significant variables
