# Supplementary material for: Application of the Andersen-Newman model of health care utilization to understand antenatal care use in Kersa District, Eastern Ethiopia
Source: PLoS One. 2018 Dec 6;13(12):e0208729. doi: 10.1371/journal.pone.0208729 (PMC6283597; doi:10.1371/journal.pone.0208729)
Supplement: S1 File — (DOCX) [file pone.0208729.s003.docx]

## Study protocol for household survey

| **Part one: Basic socio-demographic and reproductive characteristics of the participants** | | | | | | | | | | |
| --- | --- | --- | --- | --- | --- | --- | --- | --- | --- | --- |
| **1.1** | **Socio-demographic characteristics of the respondents** | | | | | | | | | |
| **No** | **Questions** | | | **Coding categories** | | | **Skip** | | **Code** | |
| Q101 | **Type of kebele** (to be completed by the interviewer) | | | 1. HDSS kebele  2. Non-HDSS kebele | | | If (2), skip to Q103 | | 1  2 | |
| Q102 | **Duration since under HDSS** (interviewer**)** | | | ---------------years | | |  | |  | |
| Q103 | **Place of residence** (to be completed by the interviewer) | | | 1.Urban  2. Rural | | |  | | 1  2 | |
| Q104 | How old are you? | | | Age in completed years | | |  | |  | |
| Q105 | What ethnic group do you belong to? | | | 1. Oromo 2. Amhara 3. Guraghe 4. Tigre   99. Other specify-------- | | |  | | 1  2  3  4  99 | |
| Q106 | What is your religion? | | | 1. Muslim 2. Orthodox 3. Catholic 4. Protestant   99. Other specify-------- | | |  | | 1  2  3  4  99 | |
| Q107 | What is your main occupation? | | | 1. Housewife 2. Farmer 3. Maidservant 4. Government employee 5. Merchant 6. Student   99. Other specify------- | | |  | | 1  2  3  4  5  6  99 | |
| Q108 | What is the highest level of formal education you have completed? | | | 1. Never attended 2. Elementary(1-8) 3. Secondary(9-12) 4. Tertiary(12^+^) | | |  | | 1  2  3  4 | |
| Q109 | What is your current marital status? | | | 1. Married 2. Divorced 3. Widowed 4. Never married 5. Separated 6. Living together (but not married) | | | If 4 or 6, skip to Q115 | | 1  2  3  4  5  6 | |
| Q110 | Age at marriage | | | ________Years | | | Except (1), for Q109, skip to Q115 | |  | |
| Q111 | In what type of marital union are you now? | | | 1. Polygamous 2. Monogamous | | | If (2), skip to Q113 | | 1  2 | |
| Q112 | Are you the first wife for your husband? | | | 1. Yes 2. No | | |  | | 1  2 | |
| Q113 | What is the highest level of formal education your husband completed? | | | 1. Never attended 2. Elementary(1-8) 3. Secondary(9-12) 4. Tertiary (12+) | | |  | | 1  2  3  4 | |
| Q114 | What is the main occupation of your husband? | | | 1. Farmer 2. Daily labourer 3. Merchant 4. Government employee   99.Other specify --------- | | |  | | 1  2  3  4  99 | |
| Q115 | Do you have any other family member in your household who received formal education? | | | 1. Yes 2. No | | | If (no), skip to 117 | | 1  2 | |
| Q116 | What is the highest level of education this person has completed? | | | 1. Elementary(1-8) 2. Secondary (9-12) 3. Tertiary (12+)   99.OtherSpecify______ | | |  | | 1  2  3  99 | |
| Q117 | Has anyone ever taught you about maternal health? | | | 1. Yes 2. No | | | If (no), skip to 119 | | 1  2 | |
| Q118 | By whom were you taught? | | | 1. HEW 2. Health care providers 3. Community health agents 4. TBA 5. Family members 6. Mass media 7. WDA leader   99.Other specify _______ | | |  | | 1  2  3  4  5  6  7  99 | |
| Q119 | Do you have any health facility in your kebele? | | | 1. Yes 2. No   88.Dont know | | |  | | 1  2  88 | |
| Q120 | Which health facility is nearest to your home? | | | 1. Health post 2. Health center 3. Hospital 4. Private clinic | | |  | | 1  2  3  4 | |
| Q121 | How do you usually get to the nearest health facility? | | | 1. On foot 2. Ambulance 3. Transport (taxi/bus) 4. Own car   99.Other specify_______ | | |  | | 1  2  3  99 | |
| Q122 | How far is the nearest health facility from your home? | | | _____in-minutes (walk)  ____in-minutes (vehicle) | | |  | |  | |
| Q123 | How do you usually get to the nearest hospital? | | | 1. On foot 2. Ambulance 3. Transport (taxi/bus) 4. Own car   99.Other specify_______ | | |  | | 1  2  3  99 | |
| Q124 | How far is the nearest hospital from your home? | | | _____in-minutes (walk)  ____ in-minutes (vehicle) | | |  | |  | |
| Q125 | Do you have the following items/resources in your household? | | | 1. Electricity 2. Wall clock 3. Radio 4. Television 5. Mobile phone 6. Refrigerator 7. Photo camera 8. DVD/CD (Video deck) 9. Bed 10. Table 11. Cabinet/cupboard 12. Bicycle 13. Motorcycle 14. Car or truck 15. Piped water source 16. Water safety means (bleaching, chlorine, water filter, boiling)? 17. Toilet facility 18. Oxen 19. Cows 20. Sheep 21. Goat 22. Donkey 23. Mules 24. Horses 25. Camels 26. Poultry 27. Own plough land 28. Bee hive 29. Charcoal/ wood stove 30. Kerosene stove 31. Sofa | | | | |  | |
| Q126 | Who is the head of the household? | | | 1. Partner 2. Respondent 3. Parents (mother/father) | | |  | | 1  2  3 | |
| Q127 | Who makes decisions about household expenses? | | | 1. Respondent 2. Partner 3. Jointly 4. Parents | | |  | | 1  2  3  4 | |
| Q128 | Has your household ever been visited by health Extension workers? | | | 1. Yes 2. No | | |  | | 1  2 | |
| Q129 | Do you have any means of communication (mass media) in your home? | | | 1. Yes 2. No | | | If (no), skip to Q131 | | 1  2 | |
| Q130 | Which means of communication (or mass media)? | | | 1. Radio 2. TV 3. Internet   99.Other specify______ | | |  | | 1  2  3  99 | |
| Q131 | Do you have a telephone (cell phone, land telephone)? | | | 1. Yes 2. No | | | If (no), skip to Q133 | | 1  2 | |
| Q132 | Does your village have a network signal for your telephone? | | | 1. Yes 2. No   88.Don’t know | | |  | | 1  2  88 | |
| Q133 | What is the birth order of the last child? | | | 1.1^st^ 2.2^nd^  3.3^rd^ 4.>3^rd^ | | | Check | | 1, 2,  3, 4 | |
| Q134 | Birth outcome for the last child | | | 1. Live full term 2. Live preterm 3. Still birth | | | Check | | 1  2  3 | |
| **1.2** | **Reproductive characteristics of the participants** | | | | | | | | | |
| Q 135 | Age of woman at first pregnancy | | | | _____Years | |  | | | |
| Q 136 | Total number of pregnancies in life time | | | |  | | Cross-check | | | |
| Q 137 | Total number of deliveries in life time | | | |  | | ” | | | |
| Q 138 | Lifetime number of livebirths | | | |  | | ” | | | |
| Q 139 | Lifetime number of stillbirths | | | |  | | ” | | | |
| Q 140 | Lifetime number of abortions (all types) | | | |  | |  | | | |
| Q 141 | Lifetime number of infant deaths (< 1 year) | | | |  | |  | | | |
| **Part two: General information about pregnancy and antenatal care** | | | | | | | | | | |
| **2.1** | **General information about pregnancy, attitude and knowledge of antenatal care** | | | | | | | | | |
| Q201 | When you became pregnant with your last birth, was that pregnancy wanted? | | 1. Yes 2. No | | | |  | | | 1  2 |
| Q202 | Have you ever heard about antenatal care services (care given to women during pregnancy)? | | 1.Yes  2. No | | | | If (no), skip to Q204 | | | 1  2 |
| Q 203 | From where did you hear about antenatal care services? | | 1. Heath facility  2. Mass media (Radio/TV)  3. TBAs  4. Community health agent  5. Relatives  6. WDA leaders  7. Other women group  99.Othersspecify_________ | | | |  | | | 1  2  3  4  5  6  7  99 |
| Q 204 | Who do you think benefits from antenatal care? | | 1. For the mother’s health 2. For the child’s health 3. Both   88. Don’t know  99. Others specify______ | | | |  | | | 1  2  3  88  99 |
| Q 205 | Do you think a healthy pregnant women should attend antenatal care clinics? | | 1. Yes 2. No | | | |  | | | 1  2 |
| Q206 | How useful do you think antenatal care is for a healthy pregnant woman? | | 1. Very useful 2. Somehow useful 3. Not useful | | | |  | | | 1  2  3 |
| Q 207 | From whom do you think a pregnant woman could get antenatal care? | | 1.Healthworker  2.TBAs  3. Community health agent  4. Relatives/friends  5. WDA leader  99.Othersspecify______ | | | |  | | | 1  2  3  4  5  99 |
| Q 208 | At what month do you think a healthy pregnant woman should first start to attend antenatal care? | | 1. 1-3months 2. 4-6months 3. 7-9months   88.Don’t know | | | |  | | | 1  2  3  88 |
| Q 209 | Are you aware of any dangerous pregnancy related symptoms? | | 1. Yes 2. No | | | | If (no), skip to Q211 | | | 1  2 |
| Q 210 | Can you mention some? (More than one answer is possible)  (Don’t read the options) | | 1. Persistent vomiting  2. Anemia  3. Leg swelling  4. Headache  5. Vaginal bleeding  6. Convulsions  7. Abnormal fetal position  8. Prolonged labor  9. Retained placenta  10. Blurred vision   1. Fever   99.Others specify_____ | | | |  | | | 1  2  3  4  5  6  7  8  9  10  11  99 |
| Q211 | If you are married, what is your husband’s attitude towards antenatal care? | | 1. Positive 2. Negative 3. Neutral   99.Don’t know | | | |  | | | 1  2  3  99 |
| Q212 | If you are married, how often did you and your husband together discuss the issue of using antenatal care during your last pregnancy? | | 1. Very often (twice monthly) 2. Often (once monthly) 3. Less often (twice during pregnancy) 4. Seldom (once during pregnancy) 5. None | | | |  | | | 1  2  3  4  5 |
| **2.2** | **Antenatal care practice and quality of service** | | | | | | | | | |
| Q213 | Did you attend antenatal care during any of your previous pregnancies before the most recent pregnancy? (Cross check) | | 1. Yes 2. No 3. Have only one pregnancy | | | |  | | | 1  2  3 |
| Q214 | Did you attend an antenatal care for your last (most recent) pregnancy? | | 1.Yes  2. No | | | | If (no) skip to Q244 | | | 1  2 |
| Q215 | Did your partner attend the antenatal care visit with you? | | 1. Yes 2. No | | | |  | | | 1  2 |
| Q216 | Who did you see for antenatal care for your last pregnancy?  (more than one answer is possible) | | 1.Doctor/health officer  2.Nurse/midwife  4.Health extension worker  99.Other-specify ________ | | | |  | | | 1  2  3  4  99 |
| Q 217 | How many months pregnant were you at your **first** antenatal checkup? | | 1. Month------   88.Don’tknow | | | |  | | | 1=0-4m  2=5-7m  3=8-9m  4=>9m  88 |
| Q 218 | How many times in total did you receive antenatal care during your pregnancy? | | 1. Once 2. Twice 3. Three times 4. Four and more | | | |  | | | 1  2  3  4 |
| Q 219 | Where did you attend your antenatal care for your last pregnancy? (more than one answer is possible) | | 1. Government hospital 2. Government health center 3. Government health post 4. Private hospital/clinics 5. Home   99. Others specify_______ | | | |  | | | 1  2  3  4  99 |
| Q 220 | What made you decide to attend your antenatal care at that particular place? (multiple response is possible)  (Don’t read the options) | | 1. Close to where you live 2. Little or no expense 3. Good service from health workers 4. Convenient time of services   99. Other specify--------- | | | |  | | | 1  2  3  4  99 |
| Q 221 | During your antenatal care visits for your last pregnancy did you ever have an injection in the arm to prevent against Tetanus? | | 1. Yes 2. No   88.Dont know | | | | If (no) skip to Q223 | | | 1  2  88 |
| Q 222 | How many times did you receive such injections? | | 1. Once 2. Two or more | | | |  | | | 1  2 |
| Q 223 | What was the main reason you started attending antenatal care? | | 1. Due to health problem 2. To start regular checkups 3. Due to my previous experience 4. To check the pregnancy 5. Due to unplanned pregnancy   99. Other specify_______ | | | |  | | | 1  2  3  4  5  99 |
| Q 224 | During any of your antenatal care visits, were you told by the health provider about signs of pregnancy complications? | | 1. Yes 2. No   88. Don’t know | | | | If (no), skip to Q226 | | | 1  2  88 |
| Q225 | Which sign of pregnancy complications were you told by the health provider? (circle all that applies)  Don’t read the options: | | 1. Vaginal bleeding 2. Heavy vaginal fluid 3. Severe headache 4. Blurred vision 5. Fever 6. Abdominal pain 7. Persistent vomiting 8. Anemia 9. Leg swelling 10. Convulsions 11. Prolonged labor   99.Other________ | | | |  | | | 1  2  3  4  5  6  7  8  9  10  11  99 |
| Q226 | Were you given information about delivering at a health facility during your antenatal care visit? | | 1. Yes 2. No | | | |  | | | 1  2 |
| Q 227 | Was blood pressure measured each time you went for antenatal care? | | 1. Always 2. On some visits 3. Never   88.Don’tknow | | | |  | | | 1  2  3  88 |
| Q 228 | Were you weighed during each antenatal care visit? | | 1. Always 2. On some visits 3. Never   88. Don’t know | | | |  | | | 1  2  3  88 |
| Q 229 | Was a laboratory examination (blood, urine, stool…) done in the antenatal care clinic? | | 1. Yes 2. Never   88. Don’t know | | | |  | | | 1  2  88 |
| Q 230 | Was a physical examination done in the antenatal care clinic during each visit? | | 1. Yes 2. No   88. Don’t know | | | |  | | | 1  2  88 |
| Q 231 | Was your height measured during the antenatal care visit? | | 1. Yes 2. No   88. Don’t know | | | |  | | | 1  2  88 |
| Q 232 | Did you ever pay for antenatal care? | | 1. Yes 2. No | | | | If (no) skip to Q235 | | | 1  2 |
| Q 233 | How did you feel about payment for antenatal care? | | 1. High 2. Moderate 3. Low 4. None | | | |  | | | 1  2  3  4 |
| Q 234 | How much on average did you pay for antenatal care service per visit? | | ___________Birr | | | |  | | |  |
| Q 235 | Do you think that the waiting time was a problem while you were attending antenatal care? | | 1. Yes 2. No   88.Don’tknow | | | |  | | | 1  2  88 |
| Q 236 | Were the health workers respectful? | | 1. Yes 2. No | | | |  | | | 1  2 |
| Q 237 | How long did you wait to get an antenatal care services? | | 1. < 2hours 2. 2-3hours 3. >3 hours | | | |  | | | 1  2  3 |
| Q 238 | Was there lack of privacy during your antenatal care? | | 1. Yes 2. No   88. Don’t know | | | |  | | | 1  2  88 |
| Q 239 | How far is the health facility where you attended antenatal care from your home? | | 1. Within 5km (very close) 2. 5-10km (manageable) 3. 10km (too far) | | | |  | | | 1  2  3 |
| Q 240 | During your antenatal care visit, were you able to ask your health provider any questions? | | 1. Yes 2. No | | | |  | | | 1  2 |
| Q241 | Did the provider tell you to come back to the facility for another visit? | | 1. Yes 2. No | | | |  | | | 1  2 |
| Q242 | Do you recommend antenatal care service to other women? | | 1. Yes 2. No | | | |  | | | 1  2 |
| Q243 | Will you attend antenatal care for your next pregnancy? | | 1. Yes 2. No 3. No plan for pregnancy | | | |  | | | 1  2  3 |
| Q244 | If you did not attend antenatal care at all for your last pregnancy, what were your reasons? (Multiple responses possible)  (Don’t read the choices) | | 1. No/little knowledge on ANC 2. Being in good health 3. Too busy to attend ANC 4. ANC service too expensive 5. Clinic too far 6. Waiting time is too long 7. Husband’s disapproval 8. No good service 9. Religious reason 10. Mistreatment by health staff in previous ANC 11. No one to accompany me   99.Others specify _______ | | | |  | | | 1  2  3  4  5  6  7  8  9  10  99 |
| **Part five: Social networking related questions** | | | | | | | | | | |
| Q301 | Is there a Women’s Development Army network in your village? | 1. Yes 2. No | | | | | | If no, skip to Q509 | |  |
| Q302 | Are you a member of the Women’s Development Army network? | 1.Yes  2.No | | | | | | If no, skip to Q508 | |  |
| Q303 | When did you become a member of the WDA network? | 1. Less than one year ago 2. Two to three years ago 3. More than 3 years ago | | | | | |  | | 1  2  3 |
| Q304 | Is there a regular WDA network meeting program in your village? | 1. Yes 2. No | | | | | | If no, skip to Q507 | | 1  2 |
| Q305 | What is the frequency of the meeting with the WDA network? | 1. Weekly 2. Fortnightly 3. Monthly 4. More than monthly | | | | | |  | | 1  2  3  4 |
| Q306 | Have you ever missed the WDA regular meeting? | 1. Yes  2. No | | | | | |  | | 1  2 |
| Q307 | Are you living in a Women’s Development Army (WDA) model family? | 1. Yes  2. No | | | | | |  | | 1  2 |
| Q308 | Does your best friend utilize/utilized any of the maternal health services (*antenatal care, delivery care or postnatal care*)? | 1.Yes  2.No  88Don’t know | | | | | |  | | 1  2  88 |
| Q309 | With whom have you had most frequent discussions about the use of any of the maternal health services? | 1. Your best friend 2. Member in the network 3. Family member 4. Member of other women group 5. No one | | | | | | If (5), skip to Q513 | | 1  2  3  4  5 |
| Q310 | Does this person use any of the maternal health services? | 1. Yes 2. No   88.Don’t know | | | | | |  | | 1  2  88 |
| Q311 | Does this person encourage you to use any of the maternal health services? | 1. Yes 2. No   88.Don’t know | | | | | |  | | 1  2  88 |
| Q312 | Who is the most influential person in another aspects of your life? | 1. Your best friend 2. Your husband 3. Relatives (mother or father) 4. Mother-in-law   99.Other specify________ | | | | | |  | | 1  2  3  4  99 |
| Q313 | Are you a member of other community-based women’s groups such as (Edir, Ekub) etc? | 1. Yes 2. No | | | | | |  | | 1  2 |
| **Part six: Social support questionnaire** | | | | | | | | | | |
| Q401 | What type of help have you received from your best friend during your pregnancy, labour and post-delivery? (*don’t mention the options but circle all that apply*) | | | | | 1.Transportation, 2.Referral  3.Material  4.Financial  5.Labour/service  6.Emotional  7.Not obtained support  99.Other specify_____ | |  | | 1  2  3  4  5  6  99 |
| Q402 | Did you get any support from the WDA network or its members? | | | | | 1. Yes 2. No | | If no, skip to Q605 | |  |
| Q403 | What kind of social support did you get from the WDA network or its members? (*more than one answer is possible*) | | | | | 1.Transportation, 2.Referral  3.Material  4.Financial  5.Labour/service  6.Emotional  99.Other specify_______ | |  | | 1  2  3  4  5  6  99 |
| Q404 | Are you satisfied with their support? | | | | | 1. Very satisfied 2. Satisfied 3. Neither 4. Dissatisfied 5. Very Dissatisfied | |  | | 1  2  3  4  5 |

*Thank you for giving your time to provide us with this information*
