# Supplementary material for: Application of the Andersen-Newman model of health care utilization to understand antenatal care use in Kersa District, Eastern Ethiopia
Source: PLoS One. 2018 Dec 6;13(12):e0208729. doi: 10.1371/journal.pone.0208729 (PMC6283597; doi:10.1371/journal.pone.0208729)
Supplement: S2 File — (DOCX) [file pone.0208729.s004.docx]

# Pirotokoolii Qorannaa ragaa manaa mantti deemamee funaanamuf

| **Kutaa tokko:** **Dhimma hawwaas--dinagdee fi wal hormaataa hirmaattotaa** | | | | | | | | | |
| --- | --- | --- | --- | --- | --- | --- | --- | --- | --- |
| **1.1** | **Dhimma hawwaas--dinagdee hirmaattotaa** | | | | | | | | |
| **Lakk** | **Gaaffilee** | | | **Qoodamiinsa** **Koodii** | | | **Irra utaali** | | **Koodii** |
| Q101 | **Gosa araddaa** (nama aaf-gaaffii gaafatunn guutama) | | | 1. HDSS araddaa  2. HDSS araddaa hin ta’in | | | Yoo (2), gara Q103 | | 1  2 |
| Q102 | **Yoom HDSS jalaseeentan** (gaafataa/ttuu**)** | | | ---------------Amata | | |  | |  |
| Q103 | **Bakka jireenya** (nama aaf-gaaffii gaafatunn guutama) | | | 1.Magaalaa  2. Baadiyaa | | |  | | 1  2 |
| Q104 | Umrii kee meeqa? | | | --------Amata guutuudhaan | | |  | |  |
| Q105 | Shanyiin kee maali? | | | 1. Oromoo 2. Amaara 3. Guraaagee 4. Tigiree 5. Kan biroo-------- | | |  | | 1  2  3  4  99 |
| Q106 | Amantaan kee maali? | | | 1. Musiliima 2. Ortodooksii 3. Kaatoolikii 4. Pheenxee/pirotestaantii   99. Kan biroo-------- | | |  | | 1  2  3  4  99 |
| Q107 | Dalagaan kee maali? | | | 1. Haadha warraa 2. Qotee bulaa 3. Hojjettuu mana namaa 4. Hojjetaa mootummaa 5. Daldala 6. Barattuu   99. Kan biroo------- | | |  | | 1  2  3  4  5  6  99 |
| Q108 | Kutaa meeeqa qaraate/batte? | | | 1. Hin baranne/qaraane 2. Kutaa(1-8) 3. Kutaa(9-12) 4. Kutaa 12 ol(12^+^) | | |  | | 1  2  3  4 |
| Q109 | Heerumteettaa? | | | 1. Heerumeera 2. Adda-baane 3. Narraa du’aa 4. Hin heerumne 5. Addaan baaneerra 6. Waliin jiraanna (garuu hin heerumne) | | | Yoo (1) hintaane, gara Q115 | | 1  2  3  4 |
| Q110 | Waggaa/amata meeqatti heerumte | | | ________Waggaa | | |  | |  |
| Q111 | Abbaaan manaa kee haadha manaa meeqa qaba? | | | 1. Lamaa fi sanaa ol 2. Tokko | | | Yoo (2) ta’e, gara Q113 | | 1  2 |
| Q112 | Ati haadha manaa jalqabaatii abbaa manaa keetiif? | | | 1. Eeyyee 2. Lakkii | | |  | | 1  2 |
| Q113 | Abbbaan manaa kee kkutaa meeqa barate? | | | 1. Hin baranne/qaraane  2. Kutaa(1-8)  3. Kutaa(9-12)  4. Kutaa 12 ol(12^+^) | | |  | | 1  2  3  4 |
| Q114 | Abbbaan manaa kee dalagaan isaa maali? | | | 1. Qotee bulaa 2. Hojjetaa guyyaa 3. Daldalaa 4. Hojjetaa mootummaa   99.Ka biroo --------- | | |  | | 1  2  3  4  99 |
| Q115 | Maatii wajjin jiraattan keesssa namni barate jiraaa? | | | 1. Eeyyee 2. Lakkii | | | Yoo (lakkii), ta’e gara 117 | | 1  2 |
| Q116 | Namni maatii kkeee keesssaaa barate kun kutaa meeqa barate? | | | 1. Hin baranne/qaraane  2. Kutaa(1-8)  3. Kutaa(9-12)  4. Kutaa 12 ol (12^+^)  99.Kan biroo______ | | |  | | 1  2  3  4  99 |
| Q117 | Waa’ee fayyaa haadholii namni si barsiise jiraa? | | | 1. Eeyyyee 2. Lakkii | | | Yoo (lakkii), ta’e gara 119 | | 1  2 |
| Q118 | Eennyutu si barsiisee? | | | 1. Hojjettoota Eksteenshinii fayyaa (HEF) 2. Ogeessa fayyaa 3. Dagaagsitoota fayyaa hawwaasa 4. Deessistuu aadaa 5. Maatii kee 6. Sabaa-himaa 7. Hoogggantuu raayyaa misooma dubatootaa   99.Kan biraa _______ | | |  | | 1  2  3  4  5  6  7  99 |
| Q119 | Araddaa keessan keesssa dhaabbati fayyaa jiraa? | | | 1. Eeyyee 2. Lakkii   88. Hinbeeku | | |  | | 1  2  88 |
| Q120 | Dhaabbata fayyaa kamtu mana keetti dhyoo dha? | | | 1. Kellaa fayyaa 2. Buufata fayyaa 3. Hospitaala 4. Kiliinika dhunfaa | | |  | | 1  2  3  4 |
| Q121 | Dhaabbata fayyaa mana keetti dhyoo akkamitti deemta? | | | 1. Miilaan 2. Ambulaansiidhaan 3. Konkolaataan 4. Konkolaataa kootiin   99.Kan biroo_______ | | |  | | 1  2  3  4  99 |
| Q122 | Dhaabbata fayyaa mana keetti dhyoo hagam fagaata? | | | _____daqiiqaa (miilaan)  ____ daqiiqaa (konkolaataa) | | |  | |  |
| Q123 | Hospitaala sitti dhihoo maaliin deemta? | | | 1. Miilaan 2. Ambulaansii 3. Konkolaataan 4. Konkolaataa kootiin   99.Kan biroo_______ | | |  | | 1  2  3  4  99 |
| Q124 | Hospitaalli sitti dhihoo hagam sirraa fagaata? | | | _____daqiiqaa (miilaan)  ____ daqiiqaa (konkolaataa) | | |  | |  |
| Q125 | Qabeenya armaan gadii kanneen mana keessa qabdaa? | | | 1. ­Elektirikii/ifaa 2. Sa’a gidaarrratti maxxanfamu 3. Raadiyoo 4. Televiijinii 5. Bilbila moobaayilii 6. Qabaneesituu ykn firiijii 7. Kaameeraa suuraa kaasu 8. Video deck 9. Sireee 10. Xarapheeszaa 11. Saaxinii waa keesssa keewwatan 12. Biskileettaa 13. Doqdoqqee 14. Konkolaataa 15. Bishaan boombaa 16. Qulqulleesituu(kiloooriinii, saamunaa) 17. Mana fincaanii 18. Sangaaa 19. Loon 20. Hoolaa 21. Re’ee 22. Harree 23. Gaangee 24. Farda 25. Gaala 26. Lukkuuu 27. Lafa qonnaa 28. Gaagura kanniisaa 29. Qoraaan/cilee 30. Naafxaa 31. kursii soofaa | | | | |  |
| Q126 | Abbaan mana kanaa eenyu? | | | 1. Naman wjjiin jiraadhu 2. Nama deebisaa jiru 3. Maatii (haadha/abbaa) | | |  | | 1  2  3 |
| Q127 | Baasii mana keessaarratti eenyutu murteessa? | | | 1. Nama deebisaa jiru  2. Naman wajjiin jiraadhu  3. waliin mari’annee  4. Maatii | | |  | | 1  2  3  4 |
| Q128 | HEF mana kee daawwattee beektii? | | | 1. Eeyyee 2. Lakkii | | |  | | 1  2 |
| Q129 | Mana kee keessatti sabaa-himaa ni hordoftaa? | | | 1. Eeyyee  2. Lakkii | | | Yoo (lakkii), ta’e gra to Q131 | | 1  2 |
| Q130 | Mana kee keessatti sabaa-himaa maal hordofta? | | | 1. Raadiyoo 2. Televijinii 3. Interneetii   99.Kan biroo______ | | |  | | 1  2  3  99 |
| Q131 | Bilbila ni qabdaa(moobaayilii/kan manaa) | | | 1. Eeyyee  2. Lakkii | | | Yoo (lakkii), ta’e gara Q133 | | 1  2 |
| Q132 | Naannoon mmana keetii neetiwoorkiin jiraa? | | | 1. Eeyyee  2. Lakkii  88.Hin beeku | | |  | | 1  2  88 |
| Q133 | Daa’imni dhumarratti dhalte daa’ima meeqafffaa dha? | | | 1.1^fffaa^ 2.2^ffaa^  3.3^ffaa^ 4.3^fffaa^ ol | | | Cheekii godhi | | 1, 2,  3, 4 |
| Q134 | Daa’imni dhumarratti dhalte akkamitti dhalate? | | | 1. Fayyaa yeroo eegeee 2. Fayyaa yeroo malee 3. Du’aa | | | Cheekii godhi | | 1  2  3 |
| **1.2** | **Haala wal hormaata nama qorannoo kana keessatti hirmaatuu** | | | | | | | | |
| Q 135 | Daa’ima jalqabaa waggaa meeqatti ulfuufte? | | | | -------Amata guutuudhaan | |  | | |
| Q 136 | Walii galtti daa’ima meeqa godhatte? | | | | ------- | | Cheekii godhi | | |
| Q 137 | Walii galattii daa’ima meeqa deesse? | | | | ------- | | “” | | |
| Q 138 | Walii galattii daa’ima meeqa fayyaa deesse? | | | | ------- | | “” | | |
| Q 139 | Walii galattii daa’ima meeqa du’aa deesse? | | | | ------- | | “” | | |
| Q 140 | Walii galattii ulfa meeqa ofirraa baafte (gosa hunda) | | | | ------- | | “” | | |
| Q 141 | Walii galattii daa’ima meeqa waaggaa osoo hinguutin sijalaa du’e | | | | ------- | | “” | | |
| **Kutaa Lama: odeeffannoo waliigalaa waa’ee ulfaa fi kunuunsa daúmsa duraa** | | | | | | | | | |
| **2.1** | **odeeffannoo waliigalaa waa’ee ulfaa, ilaalchaa fi be of antenekumsa waaée kunuunsa da’umsa duraa** | | | | | | | | |
| Q201 | Ilmoo kee kan maayyii yeroo deesse, ulfichi kan karoorfame turee? | | 1. Eyyee 2. Miti | | | |  | | 1  2 |
| Q202 | Waa’ee tajaajila kunuunsa duraa dhageessanii beektuu? | | 1.Eyyee  2. Miti | | | | Miti yoo taé, gara gaaffii  Q204 darbi | | 1  2 |
| Q 203 | Waa’ee tajaajila kunuunsa da’umsa duraa eessaa dhageessan? | | 1. Dhaabbata fayyaa  2. Sabaa himaa (Radio/TV)  3. Deessistoota aadaa  4. Dagaagsitoota Fayyaa Hawaasaa  5. Fira ykn hiriyaa  6. Gaggeessitoota garee misoomaa  7. gurmaa’insa dubartootaa biroo  99.Kanbiroo(adda baasi)_________ | | | |  | | 1  2  3  4  5  6  7  99 |
| Q 204 | Kunuunsa da’umsa duraa irraa eenyutu fayyadama jettanii yaaddu? | | 1. Fayyaa haadhaa tiif 2. F ayyaaaa daa’imaa tiif 3. lameeniifuu   88. Hin beeku  99. Kan biroo (adda baasi)______ | | | |  | | 1  2  3  88  99 |
| Q 205 | Haati ulfaa fayyaa qabdu gara dhaabbata fayyaa kunuunsa da’umsa duraaf dhaquu qabdi jettanii yaadduu? | | 1. Eyyee 2. Miti | | | |  | | 1  2 |
| Q206 | Kunuunsi da’umsa duraa haadha ulfaa fayyaa qabduu hagam fayyada jettanii yaaddu? | | 1. Baay’ee fayyada 2. Hanga tokko fayyada 3. Homaa hin fayyadu | | | |  | | 1  2  3 |
| Q 207 | Haati ulfaa kunuunsa da’umsa duraa eenyuu irraa argachuu qabdi jettanii yaaddu? | | 1.Ogeessa fayyaa  2.Deessistoota aadaa  3. Dagaagsitoota fayyaa hawaasaa  4. Fira ykn hiriyaa  5. Gaggeessitoota garee  99.Kan biroo (adda baasi)______ | | | |  | | 1  2  3  4  5  99 |
| Q 208 | Haati ulfaa fayyaa qabdu baatii meeqa irraa eegaltee kunuunsa da’umsa duraa hordofuu qabdi? | | 1. Baatii 1-3 2. Baatii 4-6 3. Baatii 7-9   88.Hin beeku | | | |  | | 1  2  3  88 |
| Q 209 | Waa’ee mallattoolee hamoo ulfaan wal qabatanii kamiinuu quba qabduu? | | 1. Eyyee 2. Miti | | | | Miti yoo ta’e, gara gaaffii  Q211 darbi | | 1  2 |
| Q 210 | Muraasa himuu dandeessuu? (Deebii tokkoo ol fudhatama qaba)  (Filannoowwan hin dubbisiiniif) | | 1. Haqqisaa wal irraa hin citne  2. hir’ina dhiigaa  3. Iita miilaa  4. Mataa bowwuu  5. Nafa saalaan dhiigni dhangala’uu  6. Hurgufuu  7.Teessuma ilmoo gadameessa keessaa sirrii hin ta’iin  8. Ciniimmuu ture  9. Obbaatiin gadameessa keessa turuu  10. Ija dura maruu   1. Dhaqna gubaa/layidaa   99.Kanbiroo(adda baasi)_____ | | | |  | | 1  2  3  4  5  6  7  8  9  10  11  99 |
| Q211 | Heerumtee yoo jiraatte ilaalchi abbaan warraa kee kunuunsa da’umsa duraa irratti qabu maal fakkaata? | | 1. Ilaalcha gaarii qaba 2. Ilaalcha gaarii hin qaba 3. Dhimma hin qabu   99.hin beeku | | | |  | | 1  2  3  99 |
| Q212 | Heerumtee yoo jiraatte yeroo ulfa kee isa maayyii abbaa manaa kee waliin waa’ee kunuunsa yeroo hagam hagamiin mari’attu? | | 1. Yeroo baay’ee (ji’atti yeroo lama) 2. Yeroo muraasa (ji’atti yeroo lama) 3. Baay’ee muraasa (waqtii ulfaatti yeroo lama) 4. Tasa (Waqtii ulfaatti yeroo tokko) 5. Gonkumaa | | | |  | | 1  2  3  4  5 |
| **2.2** | **Gocha kunuunsa da’umsa duraa fi qulqullina tajaajilichaa** | | | | | | | | |
| Q213 | Ulfa dhihoo kanaan alatti yeroo ulfa kee ammaan duraa kunuunsa da’umsa duraa hordoftee beektaa? (mirkaneeffadhu) | | 1. Eyyee 2. Miti 3. Ulfa maayyii kanaan ala hin ulfoofne | | | |  | | 1  2  3 |
| Q214 | Yeroo ulfa maayyii kunuunsa da’umsa duraa hordoftee beektaa? | | 1.Eyyee  2. Miti | | | | Miti yoo ta’e, gara gaaffii Q244 darbi | | 1  2 |
| Q215 | Abbaan manaa kee gara kunuunsa da’umsa duraa si waliin deemee beekaa? | | 1. Eyyee 2. Miti | | | |  | | 1  2 |
| Q216 | Yeroo ulfa maayyii kunuunsa da’umsa duraa irratti eenyutu si ilaale?  (deebii tokkoo ol fudhatama qaba) | | 1.Doktoora/Qondaala fayyaa  2.Nersii/Nersii deessistuu  4.Hojjettuu eksteenshinii fayyaa  99. Kanbiroo (addabaasi) __ | | | |  | | 1  2  3  4  99 |
| Q 217 | Yeroo jalqaba hordoffii kunuunsa da’umsa duraaf dhaqxu ulfi kee baatii meeqa ture? | | 1. Baatii __________   88.Hin beeku | | | |  | | 1  88 |
| Q 218 | Yeroo ulfa maayyii marsaa meeqaaf hordoffii da’umsa duraaf deemte? | | 1. Marsaa tokko 2. Marsaa lama 3. Marsaa sadii 4. Marsaa afurii fi sanaa ol | | | |  | | 1  2  3  4 |
| Q 219 | Yeroo ulfa maayyii kunuuunsa da’umsa duraa eessatti hordofte? (deebii tokkoo ol fudhatama qaba) | | 1. Hospitaala mootummaa 2. Buufata fayyaa mootummaa 3. Kellaa fayyaa 4. hospitaala/kilinika dhuunfaa 5. Manatti   99.kan biroo(adda baasi)_______ | | | |  | | 1  2  3  4  99 |
| Q 220 | Maaltu bakka itti kunuunsa argatte akka filattu si taasise? (deebisaa baay’een ni danda’ama)  (filannoo hin dubbisiniif) | | 1. Bakka jiraatutti dhihoo waan tureef 2. Baassii isaa xiqqoo ykn baasii waan hin qabneef 3. Ogeessotni fayyaa tajaajila gaarii waan kennaniif 4. Yeroon tajaajila itti kennan mijataa waan ta’eef   99. Kan biroo (adda baasi)--------- | | | |  | | 1  2  3  4  99 |
| Q 221 | Yeroo ulfa kee kan maayyii harka kee irra lilmee teetanes dirattee beektaa? | | 1. Eyyee 2. Miti   88.Hin beeku | | | | Miti yoo ta’e, gara gaaffii Q223 darbi | | 1  2  88 |
| Q 222 | Lilmee akkasii marra meeqa diratte? | | 1. Tokko 2. Lamaa fi sanaa ol | | | |  | | 1  2 |
| Q 223 | Sababni kee guddoon hordoffii da’umsa duraa itti eegalteef maali? | | 1. Rakkoo fayyaa na mudateef 2. Hordoffii walitti fufaa gochuuf 3. Muuxannoo kiyya amma duraa 4. Ulfa kiyya mirkaneeffachuuf 5. Ulfichi karooraan alatti waan ta’eef   99.Kan biroo(adda baasi)_______ | | | |  | | 1  2  3  4  5  99 |
| Q 224 | Yeroo kunuunsa da’umsa duraa waa’ee rakkoolee walxaxoo ulfaan walqabatan irratti ogeessota fayyaa irraa gorsa argattee beektaa? | | 1. Eyyee 2. Miti   88. Hin beeku | | | | Miti yoo ta’e, gara gaaffii Q226 darbi | | 1  2  88 |
| Q225 | Rakkoolee walxaxoo ulfaan walqabatan kam irratti ogeessota fayyaa irraa gorsa argatte?  (Filannoolee malan hundatti mari)  Filannoolee hin dubbisiiniif : | | 1. Nafa saalaatiin dhiigni dhangala’uu 2. Dhangala’oo hammi isaa baay’ee ta’e nafa saalaa keessaa ba’u 3. Mataa bowwoo hamaa 4. Ija dura hurrii maruu 5. Dhaqna gubaa(layidaa) 6. Dhukkubbii garaa 7. Haqqisaa wal irraa hin citne 8. Hir’ina dhiigaa 9. Iita miilaa 10. Hurgufuu 11. Ciniimmuu dheeraa   99.Kanbiroo________ | | | |  | | 1  2  3  4  5  6  7  8  9  10  11  99 |
| Q226 | Yeroo hordoffii kunuunsa da’umsa duraa dhaabbata fayyaatti da’uu akka qabdu odeeffannoo argattee? | | 1. Eyyee 2. Miti | | | |  | | 1  2 |
| Q 227 | Yeroo hordoffii kunuunsa da’umsa duraaf deddeebitu yeroo mara dhiibbaan dhiigaa kee safaramaa turee? | | 1. Yeroo hunda 2. Deddeebii tokko tokko irratti 3. Gonkumaa   88.Hin beeku | | | |  | | 1  2  3  88 |
| Q 228 | Yeroo hordoffii kunuunsa da’umsa duraaf deddeebitu yeroo mara ulfaatinni kee safaramaa turee? | | 1. Yeroo hunda 2. Deddeebii tokko tokko irratti 3. Gonkumaa   88. Hin beeku | | | |  | | 1  2  3  88 |
| Q 229 | Qorannoon laaboraatoorii (dhiigaa, fincaanii isagaraa…) yeroo hordoffii kunuunsa da’umsa duraa hojjetamaa turee? | | 1. Eyyee 2. Gonkumaa   88. Hin beeku | | | |  | | 1  2  88 |
| Q 230 | Qorannoon fayyaa qaamaa yeroo hordoffii kunuunsa da’umsa duraa hojjetamaa turee? | | 1. Eyyee 2. Miti   88. Hin beeku | | | |  | | 1  2  88 |
| Q 231 | Yeroo hordoffii kunuunsa da’umsa duraaf deddeebitu dheerinni ykn hojjaan kee safaramee turee?? | | 1. Eyyee 2. Miti   88. Hin beeku | | | |  | | 1  2  88 |
| Q 232 | Tajaajila kunuunsa duraa argatteef kafaltee beektaa? | | 1. Eyyee 2. Miti | | | | Miti yoo ta’e, gara gaaffii Q235 darbi | | 1  2 |
| Q 233 | Waa’ee kafaltii tajaajila kunuunsa da’umsa duraaf amma dura kafaltee maaltu sitti dhaga’ama? | | 1. Guddaa dha 2. Giddu galeessa 3. Xiqqaa dha 4. Homaa | | | |  | | 1  2  3  4 |
| Q 234 | Tajaajila kunuunsa da’umsa duraa tokkoof giddu galeessaan hagam kfalte? | | Qarshii __________ | | | |  | |  |
| Q 235 | Yeroon tajaajilaaf eeggachuuf turte tajaajilicha irratti rakkoo ni uuma jettee yaaddaa? | | 1. Eyyee 2. miti   88.hin beeku | | | |  | | 1  2  88 |
| Q 236 | Ogeessotni fayyaa kabajaan si keessumsiisanii? | | 1. Eyyee 2. Miti | | | |  | | 1  2 |
| Q 237 | Tajaajila kunuunsa da’umsa duraa argachuuf hagam takkaa eegde? | | 1. Sa’a 2 gadi 2. Sa’a 2-3hours 3. Sa’a 3 ol | | | |  | | 1  2  3 |
| Q 238 | Yeroo tajaajila kunuunsa da’umsa duraa argattu dhuunfummaa kan hin eegne turee? | | 1. Eyyee 2. Miti   88. Hin beeku | | | |  | | 1  2  88 |
| Q 239 | Dhaabbatni fayyaa tajaajila kunuunsa da’umsa duraa itti hordoftu mana jireenyaa kee irraa hangam fagaata? | | 1. Km 5 (baay’ee dhihoo) 2. Km 5-10 (to’atamuu danda’a) 3. Km 10 (baay’ee fagoo) | | | |  | | 1  2  3 |
| Q 240 | Yeroo kunuunsa da’umsa duraa hordofaa turte ogeessa tajaajila siif kennu gaaffii gaafachuu dandeessa turtee? | | 1. Eyyee 2. Miti | | | |  | | 1  2 |
| Q241 | Ogeessi kunuunsa siif godhaa ture beellama itti aanuuf deebitee dhufuu akka qabdu sitti himaa turee? | | 1. Eyyee 2. Miti | | | |  | | 1  2 |
| Q242 | Haati ulfaa biraan tajaajilicha akka argattu ni himtaafii? | | 1. Eyyee 2. Miti | | | |  | | 1  2 |
| Q243 | Ulfa itti aanuuf kunuunsa da’umsa duraa ni hordoftaa? | | 1. Eyyee 2. Miti 3. Ulfaa’uuf karoora hin qabu | | | |  | | 1  2  3 |
| Q244 | Yeroo ulfa maayyii garaa qabdu kunuunsa da’umsa duraa hin hordofne yoo ta’e, sababni kee maal ture? (Deebii baay’een ni danda’ama)  (Filannoolee hin dubbisiin) | | 1. Beekumsa hin qabu ykn beekumsi ani waa’ee kunuunsa da’umsa duraa irratti qabu xiqqaa dha. 2. Fayyaa gaarii qaba waan tureef 3. Yeroo hin qabun ture 4. Baasiin dhaabbilee fayyaa tajaajila kunuunsa da’umsa duraa kennanii guddaa dha. 5. Dhaabbatni fayyaa mana koo irraa fagoo dha. 6. Yeroon tajaajila argachuuf eegu dheeraa dha. 7. Abbaan manaa koo hin heyyamu 8. Tajaajilli kennamu qulqullina hin qabu 9. Amantiin kiyya hin heyyamu 10. Qabiinsi ogeessaa yeroo marsaa amma duraa waan natti hin toliiniif   99.kan biroo adda baasi _______ | | | |  | | 1  2  3  4  5  6  7  8  9  10  99 |
| **Kutaa shan: Gaaffilee network hawaasaa waliin walqabatan** | | | | | | | | | |
| Q301 | Netwok’n raayyaa misooma dubartootaa ganda kee keessa jiraa? | 1. Eyyee 2. Mit | | | | | | Miti yoo ta’e, gara gaaffii Q509 darbi |  |
| Q302 | Ati miseensa raayyaa network dubartootaa tii? | 1.Eyyee  2.Miti | | | | | | Miti yoo ta’e, gara gaaffii Q508 darbi |  |
| Q303 | Miseensa raayyaa network dubartootaa eega taatee hagam? | 1. Waggaa tokkoo gadi 2. Waggaa lamaa hanga sadii dura 3. Waggaa sadii ol | | | | | |  | 1  2  3 |
| Q304 | Raayyaa network keessanii keessatti sagantaa marii idilee qabduu? | 1. Eyyee 2. Miti | | | | | | Miti yoo ta’e, gara gaaffii Q507 darbi | 1  2 |
| Q305 | Raayyaan network keessanii yeroo hagam hagamiitti mari’attu? | 1. Torbanitti 2. Torban lama lamaan 3. Ji’aan 4. Ji’a tokkoo olitti | | | | | |  | 1  2  3  4 |
| Q306 | Marii idilee raayyaa misooma dubartootaa irraa haftee beektaa? | 1. Eyyee  2. Miti | | | | | |  | 1  2 |
| Q307 | Raayyaa misooma dubartootaa keessatti maatii adda duree keessa jiraattaa? | 1. Eyyee  2. Miti | | | | | |  | 1  2 |
| Q308 | Hiriyoota kee keessaa tajaajiloota fayyaa haawwanii ni fayyadamtii ykn fayyadamtee? (*Kunuunsa da’umsa duraa, da’umsa ykn da’umsa boodaa*)? | 1.Eyyee  2.Miti  88 hin beeku | | | | | |  | 1  2  88 |
| Q309 | Yeroo baay’ee eenyu waliin waa’ee tajaajila fayyaa haadholee irratti mari’atta? | 1. Hiriyaa kee dhihoo 2. Miseensa raayyaa network 3. Miseensa maatii 4. Miseensa jaarmaya dubartootaa biroo irraa 5. Eenyu waliinuu hin mari’adhu | | | | | | Filanno (5) yoo ta’e, gara gaaffii Q513 darbi | 1  2  3  4  5 |
| Q310 | Namni ati waliin mari’attu tajaajiloota san ni fayyadamaa? | 1. Eyyee 2. miti   88. Hin beeku | | | | | |  | 1  2  88 |
| Q311 | Namni kun akka ati tajaajiloota fayyaa haadholee kamiinuu fayyadamtu si jajjbeessaa? | 1. Eyyee 2. Miti   88.Hin beeku | | | | | |  | 1  2  88 |
| Q312 | Namni jireenya kee irratti caalmaatti dhiibbaa geessisu eenyu? | 1. Hiriyaa kee dhihoo 2. Abbaa manaa kee 3. Firoota (haadha ykn abbaa) 4. Amaatii 5. 99.kan biroo (adda baasi)________ | | | | | |  | 1  2  3  4  99 |
| Q313 | Jaarmaya hawaasaa kanneen biroo keessatti miseensummaa qabdaa (afooshaa,) etc? | 1. Eyyee 2. Miti | | | | | |  | 1  2 |
| **Kutaa jaha: Gaaffilee walgargaarsa hawaasaa** | | | | | | | | | |
| Q401 | Yeroo ulfaa, ciniimmuu fi da’umsa booda gargaarsa akkamii hiriyaa kee irraa argatte? (*filannoo hin dubbisiin garuu, deebilee malan hundattuu mari*) | | | | | 1. Geejiba, 2. Ol dabarsuu   3.Meeshaa  4.Maalaqa  5. Hojii humnaa tiin  6. Currisa/miiraan  7.Hinqebu  99.kan biroo (adda b aasi)_____ | |  | 1  2  3  4  5  67  99 |
| Q402 | Raayyaa damee misooma dubartootaa ykn miseensa isaanii irraa deggersa argattee? | | | | | 1. Eyyee 2. miti | | Miti yoo ta’e, gara gaaffii Q605 darbi |  |
| Q403 | Gargaarsa hawaasummaa akkamii raayyaa misooma dubartootaa irraa argatte?(deebii tokkoo ol ni dandaa’ama) | | | | | 1. geejiba,   2. ol erguu  3.meeshaa  4. Maalaqa  5. Hojii humnaa  6. Currisa/miiral  99. kan biroo (adda baasi_______ | |  | 1  2  3  4  5  6  99 |
| Q404 | Deggersa siif kenname irraa mirqaantee? | | | | | 1. Baay’ee itti quufeera 2. Itti quufeera 3. Homtuu natti hin dhagahamu 4. Itti hin quufne 5. Baa’ee itti hin quufne | |  | 1  2  3  4  5 |

*Yeroo kee aarsaa gootee odeeffannoo kana nuuf kennuu keetiif galatoomi*
